# A high reliability based evidential reasoning approach

**DOI:** 10.1371/journal.pone.0317438

**Published:** 2025-05-19

**Authors:** Yin Liu, Hao Li

**Affiliations:** Business School, Nanjing XiaoZhuang University, Nanjing, China; Northwestern Polytechnical University, CHINA

## Abstract

Attribute weights exert a significant effect on the solution in multi-attribute decision analysis (MADA), since solutions produced by varying attribute weights probably vary. When a decision maker has inadequate valid data, understanding or experience to produce exact attribute weights, he/she perhaps wants to seek a solution with highest reliability, referred to in this study as a highly reliable solution. To this end, a high-reliability evidential reasoning (ER) approach is put forward in the present work, which achieves alternatives comparison through determination of their reliability relative to attribute weights under ER scenario. Initially, the best alternative supported by single or multiple sets of attribute weights was determined. Then, reliability estimation is given for every alternative. In the case of highest reliability, the optimal interval of attribute weights and evaluation grades between the optimal alternative is measured and their ranking is generated. The proposed approach to the process is based on a combination of identifying these alternatives and measuring their reliability. The problem of automobile performance evaluation is explored, finding that the proposed approach is capable of effectively generating high reliability solutions for MADA problems.

## 1. Introduction

The features of decision quality, as a comprehensive concept, include professionalism, reliability, accuracy, robustness and flexibility of decision making. Decision quality can be assessed by two common approaches: evaluation of decision quality according to results; evaluation of decision quality based on process [1–3]. From one point of view, the quality of results will be affected by the quality of evaluation made by decision makers. It will also be affected by the decision makers’ confidence and satisfaction [4]. From the other point of view, quality upgrading on the process of decision making is achievable by offering decision makers the diagnostic documents and feedback [5].

In prior references, scholars have studied diverse influencing parameters of decision quality, such as quality of information [6,7], information quantity [6], way of thinking [6], decision maker quality [5,7], as well as temporal pressure [8]. For example, since corporate performance is influenced by information technology, the quality of both decision and information can be improved with the help of theory and simulation models [5]. Taking online shopping as the background, combining unconscious thinking theory with information processing theory, this paper studies how the quality of decisions is influenced by the combined effects of information quality, quantity and mode of thinking [6]. Construct rules to study how domain experts impact decision quality and to recognize experts who can make superior decisions with sophisticated expertise in the oil and gas industry [7,9]. To tackle decision making issues in economics and finance, how temporal pressure along with time-varying incentive system impact the decision quality was experimentally studied [8]. The above research is obviously domain specific and analyzes decision quality from different perspectives.

The following focuses on the generality of decision quality and the credibility of decision results in the multi-attribute decision analysis (MADA). According to the results, i.e., solutions of MADA issues, when there are great differences in the scheme performance of certain attributes, the solution to decision problem or outcomes of scheme ranking obtained in these attributes have high reliability. If a higher weight is assigned to attributes in the course of aggregation, sorting with stronger credibility will be attained after the aggregation. Hence, a factor influencing the reliability of solutions in MADA is attribute weights [10–21]. In case the attribute weights can be flexibly assigned in the MADA issue, they should be assigned for the purpose of improving the reliability of the solution [22]. Subjective method and objective method are two common methods to obtain attribute weight. In the MADA problem, the data insufficiency and inadequate comprehensive knowledge of the problem under consideration will lead the decision maker to provide inaccurate scheme ranking information. Under this scenario, generation of attribute weights from the decision matrix is possible by objective methods. However, the reliability of the solution is rarely considered in the existing objective methods.

In view of this situation, attribute weight measurement is studied to ensure that the solution of MADA is highly credible. This kind of MADA issue is impossible to solve by traditional hard computing, but can be solved by soft computing, which are a suite of methods capable of tolerating approximate reasoning, inaccuracy, as well as uncertainty. Typical soft computing techniques include probabilistic reasoning, fuzzy logic and neural computing. Evidential reasoning (ER), a soft computing technique, is crafted in the light of Dempster-Shafer theory along with decision theory, which is applied for modeling uncertainties resulting from information incompleteness or loss [23,24], and probabilistic uncertainties. Next, based on the ER method, an objective approach for attribute weight measurement is proposed, where the reliability of solution is exploited.

Assuming high data quality, sufficient time, a high level of expertise on the part of the decision maker, and an appropriate information load, this suggests that every scheme on every attribute can be rationally evaluated by decision makers. Under this hypothesis, the decision-making and subjective judgment are deemed as proper, and the scheme ranking or solution reliability is more reliant on the data fusing method. To ensure that the solution of MADA problem is highly reliable, the optimal alternative supported by single or multiple sets of attribute weights is first determined. Then the reliability measurement of each scheme is given. In the case of the highest reliability measurement, the optimal interval of attribute weights and evaluation levels between the optimal schemes is measured, and the ranking of the optimal schemes is generated.

In this paper, an evidential reasoning method based on reliability is put forward for assessing multi-attribute decision issues under three scenarios: (1) Unknown attribute weight and evaluation grade utility; (2) Unknown attribute weight and accurate evaluation grade utility; (3) Unknown rating utility and exact attribute weights. Scenarios 2 and 3 are special cases of scenario 1. In the three cases, analytical evidence reasoning algorithm is used and Min-Max regret value method construct three kinds of optimization models to determine the possible optimal scheme. Then, for each possible optimal scheme, this paper constructs three pairs of optimization intervals for generating attribute weights and evaluating grade utility. In these three cases, interval variability for each possible best scheme is defined by the attribute weight and the optimization interval of the evaluation grade utility or one of the two, which results in the ranking of possible optimal schemes.

The reminder of the present article is arranged as shown below: A literature review is given in Section 2. The ER method-related preparatory knowledge is introduced in Section 3, and is elaborated on in Section 4. The performance evaluation of six commercial vehicles is explored in Section 5, in order to prove that the ER method is applicable and effective and the proposed method are compared with the other objective methods to reveal the advantages of generating a reliable solution. Section 6 conducts a comparative analysis by numerical and simulation experiments. At last, Section 7 presents the conclusions of this study.

## 2. Literature review

In MADA, the attribute weight is an important concept. It controls how the performance of an alternative on the criterion affects the overall performance of the alternative [25,26]. As the overall performance of each alternative determines the solution to an MADA problem, attribute weights indirectly provide significant contributions to the solution. This highlights the necessity and importance of rationally determining attribute weights.

From a traditional view, attribute weights can be derived from the subjective preferences of a DM, a decision matrix, or both [27].

1. Many efforts have been made to elicit attribute weights from the subjective preferences of a DM. Representative methods include eigenvector method, linear programming model [28], point allocation [29], direct rating [30], and goal programming model based on pairwise comparison ratings [31].
2. Some studies have been conducted to determine attribute weights from a decision matrix. In the representative studies, the methods of standard deviation [32], CCSD [27], CRITIC [33], and entropy [32, 35, 36] are proposed.
3. There are other studies on determining attribute weights by synthetically employing a decision matrix and the subjective preferences of a decision maker. Representative studies include designing a two-objective mathematical programming model to integrate the subjective preferences and objective information [37], developing a general framework to integrate fuzzy preference relations, multiplicative preference relations, and a decision matrix [38], constructing a model to integrate fuzzy preferences and a decision matrix [39], and designing programming models to integrate subjective constraints on weights and a decision matrix [40].

When the subjective judgments of a decision maker on assigning weights to criteria are available, different methods may generate different attribute weights [32, 33]. As indicated by Keeney (2002)[34], attribute weights should be consistent with the true interests of a decision maker. This results in a challenge of selecting an appropriate method for generating attribute weights from subjective judgments.

In the situation where the subjective preferences of a decision maker for assigning attribute weights are partially or completely unavailable due to lack of data, knowledge, and experience, as well as to avoid personal judgment bias to the criteria, attribute weights are preferred to generate from a decision matrix or a combination of a decision matrix with partial subjective judgments. A common point in such ways to determine attribute weights is to follow the idea that the differences among the performances of all alternatives on each criterion are proportional to the weight of the criterion [27,35]. In other words, a criterion with a larger difference among the performances of all alternatives should be assigned to a larger weight. The challenges may be how to measure the difference among alternatives given different types of uncertain assessments and how to select the appropriate difference in real applications.

In addition to traditional studies, some new studies focus on the goal-specific determination of attribute weights. That is, the generation of attribute weights is intended for reaching specific goals. For example, Fu and Xu (2016) [41] proposed a method of determining attribute weights to achieve solution with high reliability. Dong et al. (2018) [42] discussed how to help a dishonest decision maker assign strategic attribute weights to obtain his or her desired rankings of alternatives. These studies are closely associated with specific decision goals.

## 3. Preliminaries

### 3.1. ER approach to modeling MADA problems

Since attribute weights are intended for MADA issues under ER scenario, the ways of modeling and assessing MADA issues under ER scenario are presented as shown below.

Assuming *M* alternatives *a*_*l*_ (*l* = 1, …, *M*) along with *L* attributes *e*_*i*_ (*i* = 1, …, *L*) are present in a MADA issue. For *L* attributes, their relative weights are signified by *w* = (*w*_1_, *w*_2_, …, *w*_*L*_), enabling 0 ≤ *w*_*i*_ ≤ 1 and $\sum {\mathrm{\backslash nolimits}}_{i=1}^{L}w_{i}$ = 1.

A decision maker can determine a grade set *Ω* = {*H*_1_, *H*_2_, …, *H*_*N*_}, which is sorted in an ascending order of quality, so that every alternative on every standard is assessable in the ER context. Semantics of grade *H*_*n*_ are described through utilities settings for assessment grades *u*(*H*_*n*_) (*n* = 1, …, *N*), which fulfill constraint 0 = *u*(*H*_1_) < *u*(*H*_2_) < … < *u*(*H*_*N*_) = 1. Suppose that the evaluation of alternative *a*_*l*_ on standard *e*_*i*_ is profiled using a belief distribution *B*(*e*_*i*_(*a*_*l*_)) = {(*H*_*n*_, *β*_*n*,*i*_(*a*_*l*_)), *n* = 1, …, *N*; (*Ω*, *β*_*Ω*,*i*_(*a*_*l*_))}, with *β*_*n*,*i*_(*a*_*l*_) ≥ 0, $\sum {\mathrm{\backslash nolimits}}_{n=1}^{N}\beta_{n,i}(a_{l})$ ≤ 1, and *β*_*Ω*,*i*_(*a*_*l*_) = 1 − $\sum {\mathrm{\backslash nolimits}}_{n=1}^{N}\beta_{n,i}(a_{l})$. In the distribution, *β*_*n*,*i*_(*a*_*l*_) refers to the belief degree allocated to grade *H*_*n*_; *β*_*Ω*,*i*_(*a*_*l*_) stands for the global ignorance degree. The evaluation is complete in case *β*_*Ω*,*i*_(*a*_*l*_) = 0, while is incomplete in other cases. Where *B*(*e*_*i*_(*a*_*l*_)) (*i* = 1, …, *L*, *l* = 1, …, *M*) is designated, a belief decision matrix *S*_*L*×*M*_ can be acquired.

Through using attribute weights along with ER rule, the total assessment *B*(*y*(*a*_*l*_)) = {(*H*_*n*_, *β*_*n*_(*a*_*l*_)), *n* = 1, …, *N*; (*Ω*, *β*_*Ω*_(*a*_*l*_))} is derived by integrating individual assessments *B*(*e*_*i*_(*a*_*l*_)). Here, *β*_*Ω*_(*a*_*l*_) symbolizes the total global ignorance degree. Next, by integrating total assessment and grade utilities, the anticipated minimum and maximum utilities were derived for alternative *a*_*l*_ as


$$\begin{array}{c} u-\left(al\right)\;=\sum {\mathrm{\backslash nolimits}}_{n=2}^{N}\beta_{n}(a_{l})\cdot u(H_{n})\;+\;\left(\beta 1\left(al\right)+\beta \Omega \left(al\right)\right)\cdot u\left(H1\right)\text{ and} \end{array}$$

$$\begin{array}{c} u+\left(al\right)=\;\sum {\mathrm{\backslash nolimits}}_{n=1}^{N-1}\beta_{n}(a_{l})\cdot u(H_{n})+\left(\beta N\left(al\right)+\beta \Omega \left(al\right)\right)\cdot u\left(HN\right). \end{array}$$

In the light of decision rules like the minimax regret rule or Hurwicz rule, the *M* alternatives can be compared from [*u*^−^(*a*_*l*_), *u*^+^(*a*_*l*_)], thereby deriving a solution to the MADA issue. Especially, when the individual ignorance on each criterion is assigned by following some way, the expected utility of each alternative reduces to a precise number and a solution can be directly made from the expected utilities of alternatives.

### 3.2. Measurement of reliability of aggregated solution

Following the obtainment of minimal satisfaction of alternative *V*(*a*_*l*_), the aggregated solution reliability is presented as follows by refer to relevant concepts of Fu and Xu [41].

**Definition 1.** [41] Assuming that *V*(*b*_*l*_) (*l* = 1, …, *M*) stands for the ordered least satisfaction of alternatives enabling *V*(*b*_1_) ≥ … ≥ *V*(*b*_*m*_). In other words, *V*(*b*_*l*_) indicates the *l*th greatest of *V*(*a*_1_), …, *V*(*a*_*m*_). Accordingly, for alternative *b*_*l*_ (*l* = 1, …, *M* – 1), ∆*V*(*b*_*l*_), its superior intensity is expressed as ∆*V*(*b*_*l*_) = $\sum {\mathrm{\backslash nolimits}}_{m=l+1}^{M}(V(b_{l})-\;V(b_{m}))/2$ (*l* = 1, …, *M* − 1), while the formula for aggregated solution reliability is *Q* = $\sum {\mathrm{\backslash nolimits}}_{l=1}^{M-1}\phi_{l}\cdot △V(b_{l})$, with *ϕ*_*l*_ (*l* = 1, …, *M* − 1) denoting the weights of ∆*V*(*b*_*l*_) (*l* = 1, …, *M* − 1) for *Q* that enables 0 ≤ *ϕ*_*l*_ ≤ 1 (*l* = 1, …, *M* − 1) and $\sum {\mathrm{\backslash nolimits}}_{l=1}^{M-1}\phi_{l}=1$.

Definition 1 unveils the establishment of aggregated solution reliability as the weighted mean for the alternative's superior intensity. Explanations on properties associated with ∆*V*(*b*_*l*_) (*l* = 1, …, *M* − 1) and *Q* are shown below.

Property 1. [41] Given ∆*V*(*b*_*l*_) (*l* = 1, …, *M* − 1) and *Q* in Definition 1, the following relationships hold:


\[ΔV(b1) ≥ … ≥ ΔV(bM −1),\] (1)

$$\mathrm{\backslash [}\Delta V\left(b_{x}\right)\;=\;\ldots \;=\Delta V\left(b_{y}\right),\;ifV\left(b_{x}\right)\;=\;\ldots \;=V\left(b_{y}\right)\;forx<y\;and\;x,y\;\{1,\;\ldots ,M-1\}\mathrm{\backslash ]}$$ (2)

$$\mathrm{\backslash [}0\leq \Delta V\left(b_{l}\right)\leq M-l,\;and\mathrm{\backslash ]}$$ (3)

0≤Q≤M−1. (4)

Assumption 1 [41] Assuming *ϕ*_*l*_ (*l* = 1, …, *M* − 1) denotes the weight of ∆*V*(*b*_*l*_) (*l* = 1, …, *M* − 1) for *Q* in Definition 1. In order to underline the declining contribution of ∆*V*(*b*_*l*_) (*l* = 1, …, *M* − 1) to *Q*, the following is subsequently required


ϕ1> …> ϕM−1 > 0, (5)

\[(ϕl − ϕl−1) − (ϕl+1 − ϕl+2) = dl − dl+1 = Δd > 0 (l = 1, …, M − 3),\] (6)

$$and\;\sum {\mathrm{\backslash nolimits}}_{l=1}^{M-1}\phi_{l}=1$$ (7)

Based on Assumption 1, *ϕ*_*l*_ (*l* = 1, …, *M* − 1) is estimable in case the greatest weight *ϕ*_1_ is designated.

**Theorem 1.** [41] Assuming *ϕ*_*l*_ (*l* = 1, …, *M* − 1) denotes the weight of ∆*V*(*b*_*l*_) (*l* = 1, …, *M* − 1) for *Q* and ∆*d* in Definition 1. Where the greatest weight *ϕ*_1_ is designated, *ϕ*_*l*_ (*l* = 2, …, *M* − 1) is calculated using *ϕ*_1_ − (*l* − 1)*d*_1_ + $\frac{\left(l-2cdot(l-1\right)}{2}$∆*d* by the minimax disparity approach exploiting Assumption 1. That is, the greatest disparity of *ϕ*_*l*_ from *ϕ*_*l*+1_ (*l* = 1, …, *M* − 2) is minimized, with *d*_1_ = $\frac{4(M-1)\phi_{1}-6}{\left(M-2)(M-1\right)}$ + *ε*, ∆*d* = $\frac{6(M-1)\phi_{1}-12}{\left(M-3)(M-2)(M-1\right)}$ + $\frac{3\varepsilon }{M-3}$, and *ε* being a tiny positive number that approximates 0.

With the purpose of ensuring the validity of Theorem 1, the following theorem was exploited to ascertain the permissible ranges of *ϕ*_1_.

**Theorem 2.** [41] Suppose that *ϕ*_*l*_ (*l* = 1, …, *M* − 1) denotes the weight of ∆*V*(*b*_*l*_) (*l* = 1, …, *M* − 1) for *Q* in Theorem 1, the calculation of *ϕ*_*l*_ (*l* = 2, …, *M* − 1) in Theorem 1 following Assumption 1 requires that $\frac{4-(M-2)(M-1varepsilon}{2(M-1)}$ < *ϕ*_1_ < $\frac{3-(M-2)(M-1varepsilon}{M-1}$, with *ε* being a tiny positive number that approximates 0.

For the monotonicity assessment of *Q* relative to *ϕ*_1_ during *Q* maximization, the relationship between *Q* and *ϕ*_1_ are investigated using the theorem below.

**Theorem 3.** [41] Let aggregated solution reliability be *Q* = $\sum {\mathrm{\backslash nolimits}}_{l=1}^{M-1}\phi_{l}\cdot △V(b_{l})$, with 0 ≤ *ϕ*_*l*_ ≤ 1 (*l* = 1, …, *M* − 1) and $\sum {\mathrm{\backslash nolimits}}_{l=1}^{M-1}\phi_{l}=1$, *ϕ*_1_ fulfills requisites provided in Theorem 2, and ∆*V*(*b*_*l*_) = $\sum {\mathrm{\backslash nolimits}}_{m=l+1}^{M}(V(b_{l})-\;V(b_{m}))/2$ (*l* = 1, …, *M* − 1), *Q* shows monotonous elevation respecting *ϕ*_1_.

## 4. The proposed method based on the reliability of the aggregated solution

**Fig 1:**
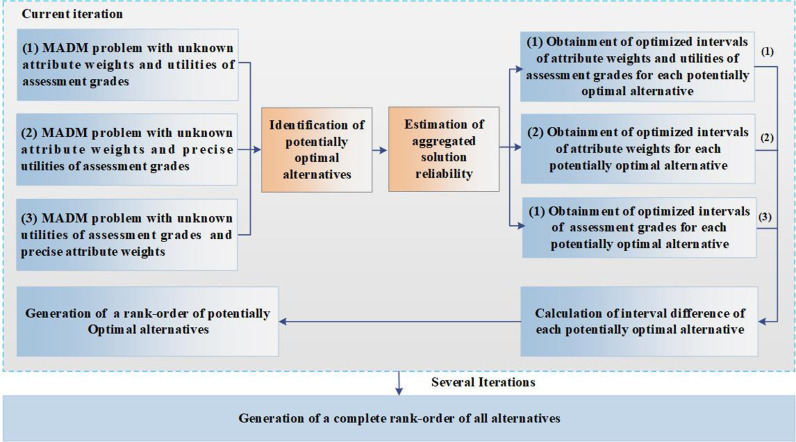
The approach applied to analyzing the problems.

In the present section, we present our proposed method. The problems addressed in this paper and the approach applied to analyzing the problems are summarized in Fig 1. Potentially best alternatives are identified by creating an optimization model, which is incorporated with subjective constrains on attribute weights and evaluation grade utilities. Then, the interval of aggregated solution reliability is estimated for the identified optimal alternatives by creating another optimization model. After that, interval determination for attribute weights and evaluation grade utilities is accomplished in the interval of aggregated solution reliability for the identified optimal alternatives. Through considering synthetically with the obtained three intervals, generation of a ranking order is possible for the entire identified optimal alternatives.

### 4.1. Identification of potentially optimal alternatives

As demonstrate in Section 3.1, with the known *w* and the specified *u*(*H*_*n*_) (*n* = 1, …, *N*), the least satisfaction of alternatives *V*(*a*_*l*_) (*l* = 1, …, *M*) is obtainable from the evaluations *B*(*e*_*i*_(*a*_*l*_))_*L*×*M*_, yielding a sole solution for a MADA issue. On the downside, under some circumstances, *w* and *u*(*H*_*n*_) (*n* = 1, …, *N*) maybe completely unknown or partially available. Multiple alternatives may have the minimal satisfaction and be identified as the optimal alternative. To cope with the situation where a possible set of optimal alternatives may exist due to the fully unspecified or partially available of attribute weights and evaluation grade utilities, the potentially best alternatives are determined by creating an optimization model.


$$\begin{array}{c} \text{MAX}V\left(al\right)-\max_{m\in \{1,...,M\}}\{V(a_{m})\}\;\left(l=1,\ldots ,M\right) \end{array}$$ (8)

$$\begin{array}{c} \text{s.t.}V\left(al\right)=\;u-\left(al\right)-maxm\;\text{1}\;l\left\{u\;+\;\left(am\right)\right\},l=\;1,\ldots ,M, \end{array}$$ (9)

$$\mathrm{\backslash [}u-\left(al\right)\;=\sum {\mathrm{\backslash nolimits}}_{n=2}^{N}\beta_{n}(a_{l})\cdot u\;(H_{n})\;+\;\left(\beta 1\left(al\right)\;+\;\beta \;\Omega \;\left(al\right)\right)\cdot u\left(H1\right)\mathrm{\backslash ]}$$ (10)

$$\mathrm{\backslash [}u+\left(al\right)\;=\;\sum {\mathrm{\backslash nolimits}}_{n=1}^{N-1}\beta_{n}(a_{l})\cdot u(H_{n})+\;\left(\beta N\left(al\right)\;+\;\beta \;\Omega \left(al\right)\right)\cdot u\left(HN\right)\mathrm{\backslash ]}$$ (11)

$$\sum {\mathrm{\backslash nolimits}}_{i=1}^{L}w_{i}(a_{l})=1,$$ (12)

$$\mathrm{\backslash [}w_{i}^{r}\;\leq \;w_{i}^{-}\;\leq \;wi\left(al\right)\;\leq w_{i}^{+},\;i\;=\;1,\;\ldots ,\;L,\mathrm{\backslash ]}$$ (13)

$$wi(al,\in G_{w},\;i\;=\;1,\;\ldots ,\;L,$$ (14)

0 = u(H1\rightleft(al) < u(H2)(al) <… < u(HN)(al) = 1, (15)

u(Hn\rightleft(al) − u(Hn−1)(al) ≥ δu, n = 2, …, N. (16)

u−(Hn)£u(Hn\rightleft(al)£u+(Hn),n=1,…,N, (17)

u(Hn\rightleft(al), i = 1, …, L, (18)

In the above model, the parameters $w_{i}^{r}$, $w_{i}^{-}$, $w_{i}^{+}$, *δ*_*u*_, *u*^−^(*H*_*n*_) and *u*^+^(*H*_*n*_) are designated by the decision maker, with $w_{i}^{r}$ being the constraints on $w_{i}^{-}$, which used to control each attribute to contribute to a solution. $w_{i}^{-}$ and $w_{i}^{+}$ denote inferior and superior limits of *w*_*i*_(*a*_*l*_) for a potentially best alternative *a*_*l*_. *u*^−^(*H*_*n*_) and *u*^+^(*H*_*n*_) refer to inferior and superior limits of *u*(*H*_*n*_)(*a*_*l*_). *δ*_*u*_ represents the constraint on least disparity of *u*(*H*_*n*_)(*a*_*l*_) from *u*(*H*_*n*−1_)(*a*_*l*_). *w*_*i*_(*a*_*l*_) (*i* = 1, …, *L*; *l* = 1, …, *M*) and *u*(*H*_*n*_)(*a*_*l*_) (*n* = 1, …, *N*; *l* = 1, …, *M*) stand for decision variables in the intervals.

According to the minimax regret approach (MRA) [38], the smaller *R*(*a*_*l*_), the better an alternative *a*_*l*_ compared with the others. Thus, if the optimized objective of an alternative *a*_*l*_ in Eq. (8) is equal to 0, *R*(*a*_*l*_) is the smallest; that is, the alternative al can become the optimal. In Eqs. (10) and (11), *u*^*−*^(*a*_*l*_) and *u*^+^(*a*_*l*_) integrate *V*(*a*_*l*_) plus *β*_*Ω*_(*a*_*l*_) with *u*(*H*_*n*_)(*a*_*l*_) (*n* = 1, …, *N*, *l* = 1, …, *M*) to form *V*(*a*_*l*_) in Eq. (9), which can produce a ranking order of alternative. In Eq. (17), *u*(*H*_*n*_)(*a*_*l*_) is limited to [*u*^−^(*H*_*n*_), *u*^+^(*H*_*n*_)]. The intrinsic constraint on *u*(*H*_*n*_)(*a*_*l*_) is expressed as 0 = *u*(*H*_1_)(*a*_*l*_) < *u*(*H*_2_)(*a*_*l*_) < … < *u*(*H*_*N*_)(*a*_*l*_) = 1 in Eq. (15). The minimal difference between *u*(*H*_*n*_)(*a*_*l*_) and *u*(*H*_*n*−1_)(*a*_*l*_) is limited by *δ*_*u*_ in Eq. (16). In Eq. (13), *w*_*i*_(*a*_*l*_) is limited to [$w_{i}^{-}$, $w_{i}^{+}$] and $w_{i}^{r}$ ≤ $w_{i}^{-}$ is needed. Eq. (14) shows the constraint on *w*_*i*_(*a*_*l*_) (*i* = 1, …, *L*). The alternatives with the zero−valued optimized objectives in the above model are called potentially optimal alternatives, which form *M*_*b*_.

In Eq. (9), exploiting nonlinear analytical algorithm, the evaluations *B*(*y*(*a*_*l*_)) (*l* = 1, …, *M*) are generated through summation of evaluations *B*(*e*_*i*_(*a*_*l*_))_*L*×*M*_ weighted by *w*_*i*_(*a*_*l*_), and through their further summation, the anticipated minimal and maximal utilities *u*^−^(*a*_*l*_) and *u*^+^(*a*_*l*_) (*l* = 1, …, *M*) with *u*(*H*_*n*_)(*a*_*l*_) are generated, as displayed in Section 3.1. With *u*^−^(*a*_*l*_) and *u*^+^(*a*_*l*_), computation of least satisfaction is accomplished for alternative *V*(*a*_*l*_) (*l* = 1, …, *M*). The normalized constraint on *w* is expressed in Eq. (10). In Eq. (12), the trait of partially available attribute weights information is a range of constraints on *w*, which are represented by *G*_*w*_. Table 1 lists the six possible constraint types in *G*_*w*_. The inherent constraint on *u*(*H*_*n*_)(*a*_*l*_) is denoted in Eq. (13). Similarly to Eq. (12), in Eq. (16), the partially available information on utilities of assessments grades are typified by a range of constraints on *u*(*H*_*n*_)(*a*_*l*_) and represented by *G*_*u*_. Table 2 lists the five possible constraint types in *G*_*u*_. Hereinto, *p*_23_ and *r*_23_ refer separately to the reciprocal fuzzy and multiplicative preference associations, whose value intervals are [0, 1] and [1/9, 9].

**Table 1:** The different types of constraints on attribute weights.

	Types of constraints	Form
Form 1	Pairwise constraint	*w*_*i*_ > *w*_*j*_
Form 2	Preference constraint intensity	*w*_*i*_ − *w*_*j*_ > *w*_*k*_− *w*_*l*_
Form 3	Bounded constraint	*LB*_*i*_ ≤ *w*_*i*_ ≤ *UB*_*i*_
Form 4	Bounded preference ratio constraint	*LB*_*i*_ ≤ *w*_*i*_/*w*_*j*_ ≤ *UB*_*i*_
Form 5	Bounded preference difference constraint	*LB*_*i*_ ≤ *w*_*i*_ − *w*_*j*_ ≤ *UB*_*i*_
Form 6	Multivariate linear inequality constraint	*w*_*i*_ + *w*_*j*_ > *w*_*k*_

**Table 2:** The different types of constraints on utilities of assessments grades.

	Types of constraints	Form
Form 1	Fuzzy preference constraint	*u*(*H*_2_)/(*u*(*H*_2_) + *u*(*H*_3_)) > *p*_23_
Form 2	Multiplicative preference constraint	*u*(*H*_2_)/*u*(*H*_3_) = *r*_23_
Form 3	Linear inequality constraint	*u*(*H*_2_) + *u*(*H*_3_) ≥ *u*(*H*_4_)
Form 4	Fuzzy preference inequality constraint	*u*(*H*_2_)/(*u*(*H*_2_) + *u*(*H*_3_)) ≥ *p*_23_
Form 5	Multiplicative preference inequality constraint	*u*(*H*_2_)/*u*(*H*_3_) ≥ *r*_23_

According to the definition of minimal satisfaction in Section 3.1, greater preference is given to the alternative *a*_*l*_ with higher minimal satisfaction. Hence, the alternative *a*_*l*_ is identified as the potentially optimal alternative if and only if the optimized purpose of alternative *a*_*l*_ in Eq. (8) is equivalent to 0. In other words, *a*_*l*_ is identified as the potentially best alternative if the minimal satisfaction of alternative al is largest in comparison with those of other alternatives. Suppose that *M*_*b*_ denotes the set of potentially best alternatives. The alternatives whose optimized objective values are 0 in the foregoing model forms *M*_*b*_.

### 4.2. Determination of the intervals of reliability of the aggregated solution for the identified optimal alternatives

After all the potentially optimal alternatives are identified and included in *M*_*b*_, the issue left is further comparison of alternatives in *M*_*b*_ if more than one alternative is identified as potentially optimal alternative. In particular, if only one alternative in *M*_*b*_, the one is identified as the optimal alternative. In theory, the alternatives in *M*_*b*_ correspond to different solutions to MADA problems. In terms of the decision quality of different solutions, more credible solution is often favored by the decision maker when making decisions [41]. To guarantee the solution in line with the preference of decision maker, it may be feasible to measure the aggregated solution reliability for the alternatives in *M*_*b*_, thus achieving reliability comparison among alternatives. On the other hand, an alternative in *M*_*b*_ may correspond to multiple sets of attribute weights along with assessments grade utilities. Aggregated solution reliability for the alternatives in *M*_*b*_ may vary along with the fluctuations of attribute weights, as well as assessments grade utilities. To reflect the solution reliability robustness of alternatives in *M*_*b*_ to the fluctuations of attribute weights and assessments grade utilities, the possible intervals of aggregated solution reliability for the alternatives in *M*_*b*_ is measured, which is elaborated as follows.

Suppose that *Q*(*a*_*l*_) denotes the aggregated solution reliability for the alternative *a*_*l*_ when *a*_*l*_ is identified as the potentially optimal alternative. Then, *Q*^−^(*a*_*l*_) and *Q*^*+*^(*a*_*l*_) respectively stand for the lowest and highest reliabilities of aggregated solution for the alternative *a*_*l*_, which are estimated by addressing a pair of nonlinear optimization issues shown below:


$$\begin{array}{c} \text{Max/Min}\;\;Q(a_{l})=\sum {\mathrm{\backslash nolimits}}_{l=1}^{M-1}\phi_{l}\cdot △V(b_{l}) \end{array}$$ (19)

$$\begin{array}{c} \text{s.t. }V\left(al\right)\;-\max_{m\in \{1,...,M\}}\{V(a_{m})\}=\;0,\;a_{l}\in M_{b}, \end{array}$$ (20)

To evade repetition, we elide the constraints in Eqs. (9)–(18) in the foregoing optimization models in section 4.1. The calculation of *Q*(*a*_*l*_) is demonstrated in Definition 1. In theory, *Q*(*a*_*l*_) is considered as a continuous function associated with the variables of *w*_*i*_(*a*_*l*_) (*i* = 1, …, *L*; *l* = 1, …, *M*), as well as *u*(*H*_*n*_)(*a*_*l*_) (*n* = 1, …, *N*; *l* = 1, …, *M*). Besides, maximization of *Q*(*a*_*l*_) is closely associated with the *ϕ*_*l*_ (*l* = 1, …, *M* − 1). As is shown in Theorem 3, *Q*(*a*_*l*_) monotonously increasing with respect to *ϕ*_1_. In other words, *Q*(*a*_*l*_) value is greatest if and only if maximal *ϕ*_1_ is achieved, while minimum *Q*(*a*_*l*_) is reached if and only if *ϕ*_1_ is minimized. In order to obtain the maximum *Q*(*a*_*l*_), the maximal *ϕ*_1_ should be guaranteed. On the contrary, to obtain the minimum *Q*(*a*_*l*_), the minimal *ϕ*_1_ should be guaranteed. On the assumption that the maximum *ϕ*_1_ and minimum *ϕ*_1_ are determined by using Theorem 2, the intervals of reliability of the aggregated solution for the identified optimal alternatives are assessable by addressing the foregoing pair of optimization issues. In the ER approach, an alternative with high reliability is better than others. To facilitate the comparison of alternatives, we introduce the reliability into the foregoing pair of optimization issues, which is opposed to the maximal regret. According, an alternative with larger reliability is better than the others. From Property 1, we can infer that [*Q*^−^(*a*_*l*_), *Q*^*+*^(*a*_*l*_)] belongs to [0, *M* − 1]. For the alternative in *M*_*b*_, an aggregated solution with larger reliability intervals is more favored by decision maker.

### 4.3. Determination of the intervals of attribute weights and utilities of assessment grades for the identified optimal alternatives

In the present section, attribute weights and evaluation grader utilities for identified optimal alternatives will be determined under the condition of making the aggregation solution most reliable. Interval attribute weights are determined for each alternative by creating three optimization problem pairs, such that al ∈ Mb and the intervals for utilities of assessments grades, as described below.

To determine [${\text{w}}_{i}^{-}(a_{l})$, ${\text{w}}_{i}^{\text{+}}(a_{l})$] enabling al ∈ Mb in case one, an optimization problem pair is established as shown below:


$$\begin{array}{c} \text{Max/Min }wi\left(al\right) \end{array}$$ (21)

\[s.t. Q+(al) - φ £Q(al)\] (22)

$$V\left(a_{l}\right)-\max_{m\in \{1,...,M\}}\{V(a_{m}\}=\;0,\;a_{l}\in M_{b},$$ (23)

$$\sum {\mathrm{\backslash nolimits}}_{i=1}^{L}w_{i}(a_{l})=1,$$ (24)

$$\mathrm{\backslash [}w_{i}^{r}\;\leq \;w_{i}^{-}\;\leq \;wi\left(al\right)\;\leq w_{i}^{+},\;i\;=\;1,\;\ldots ,\;L,\mathrm{\backslash ]}$$ (25)

$$wi(al,\in G_{w},\;i\;=\;1,\;\ldots ,\;L,$$ (26)

0 = u(H1\rightleft(al) < u(H2)(al) <… < u(HN)(al) = 1, (27)

u(Hn\rightleft(al)−u(Hn−1)(al)3δu,n=2,…,N. (28)

u−(Hn)£u(Hn\rightleft(al)£u+(Hn),n=1,…,N, (29)

$$u\left(Hn\mathrm{\backslash rightleft}(al\right)\in G_{u},\;i\;=\;1,\;\ldots ,\;L,$$ (30)

The constraint in Eq. (23) is computed by an identical procedure to that followed for objective function computation in Eq. (8), which is therefore elided from the foregoing optimization problem. After the model shown in Eqs. (19)–(20) is solved, its optimal objectives denoted by *Q*^−^(*a*_*l*_) and *Q*^*+*^(*a*_*l*_) can be obtained. The optimal objective is definitely accompanied with a set of criterion weights and utilities of grades. The set of criterion weights and utilities of grades may not be unique. There may be other sets of criterion weights and utilities of grades leading to the same objective values *Q*^−^(*a*_*l*_) and *Q*^*+*^(*a*_*l*_). To cover all possible sets of criterion weights and utilities of grades resulting in *Q*^−^(*a*_*l*_) and *Q*^*+*^(*a*_*l*_) , a slightly enlarged post−optimal solution space is defined as *Q*^+^(*a*_*l*_) − *φ* ≤ *Q*(*a*_*l*_) for further analysis, where *φ* is a very small positive number such as 0.001 to compensate computational error incurred in the process of generating *Q*(*a*_*l*_). In case objective function in Eq. (21) becomes *u*(*H*_*n*_)(*a*_*l*_), we can get [*u*^*−*^(*H*_*n*_)(*a*_*l*_), *u*^*+*^(*H*_*n*_)(*a*_*l*_)] by seeking best solution of the foregoing optimization problem. In general, the larger range of *w*_*i*_(*a*_*l*_) and *u*(*H*_*n*_)(*a*_*l*_) of the alternative *a*_*l*_ is with the others, the more robust a solution is.

Under scenario two, another optimization problems pair is created for evaluating [${\text{w}}_{i}^{-}(a_{l})$, ${\text{w}}_{i}^{\text{+}}(a_{l})$], which enables al ∈ *M*_*b*_.


$$Max/Min\;w_{i}\left(a_{l}\right)$$ (31)

\[s.t. Q+(al) − φ ≤ Q(al)\] (32)

$$\mathrm{\backslash [}V\left(al\right)\;-\;\;\max_{m\in \{1,...,M\}}\{V(a_{m})\}=0,\;a_{l}\in M_{b},\mathrm{\backslash ]}$$ (33)

$$\sum {\mathrm{\backslash nolimits}}_{i=1}^{L}w_{i}(a_{l},=\;1,$$ (34)

$$\mathrm{\backslash [}w_{i}^{r}\;\leq \;w_{i}^{-}\;\leq \;wi\left(al\right)\;\leq w_{i}^{+},\;i\;=\;1,\;\ldots ,\;L,\mathrm{\backslash ]}$$ (35)

$$wi(al,\in G_{w},\;i\;=\;1,\;\ldots ,\;L,$$ (36)

The calculation of the constraint in Eq. (33) is the same as the objective function in Eq. (8). It is thus omitted in the above optimization model. After the model shown in Eqs. (19)–(20) is solved, its optimal objectives denoted by *Q*^−^(*a*_*l*_) and *Q*^*+*^(*a*_*l*_) can be obtained. The optimal objective is definitely accompanied with a set of criterion weights. The set of criterion weights may not be unique. There may be other sets of criterion weights leading to the same objective values *Q*^−^(*a*_*l*_) and *Q*^*+*^(*a*_*l*_). To cover all possible sets of criterion weights resulting in *Q*^−^(*a*_*l*_) and *Q*^*+*^(*a*_*l*_) , a slightly enlarged post−optimal solution space is defined as *Q*^+^(*a*_*l*_) − *φ* ≤ *Q*(*a*_*l*_) for further analysis, where *φ* is a very small positive number such as 0.001 to compensate computational error incurred in the process of generating *Q*(*a*_*l*_).

Under scenario three, another optimization problems pair is created for evaluation of [*u*^*−*^(*H*_*n*_)(*a*_*l*_), *u*^*+*^(*H*_*n*_)(*a*_*l*_)].


$$Max/Min\;u\left(H_{n}\mathrm{\backslash rightleft}(a_{l}\right)$$ (37)

\[s.t. Q+(al) − φ ≤ Q(al)\] (38)

$$\mathrm{\backslash [}V\left(al\right)\;-\max_{m\in \{1,...,M\}}\{V(a_{m})\}\;=0,\;a_{l}\in M_{b}\;=0,\;\mathrm{\backslash ]}$$ (39)

0 = u(H1\rightleft(al) < u(H2)(al) <… < u(HN)(al) = 1, (40)

u(Hn\rightleft(al) − u(Hn−1)(al) ≥ δu, n = 2, …, N. (41)

\[u−(Hn) ≤ u(Hn)(al) ≤ u+(Hn), n = 1, …, N,\] (42)

$$u\left(Hn\mathrm{\backslash rightleft}(al\right)\in G_{u},\;i\;=\;1,\;\ldots ,\;L,$$ (43)

In the foregoing three optimization issue pairs, subjective restrictions on *u*(*H*_*n*_) (*n* = 1, ..., *N*) and *w* are manageable as well. An example of this is presented in Section 6 below, where restrictions on *u*(*H*_*n*_) (*n* = 1, ..., *N*) are dealt with.

### 4.4. Generation of a ranking order of potentially optimal alternatives

For above mentioned three scenarios in Section 4.3, the interval difference enabling *a*_*l*_ ∈*M*_*b*_ for an alternative *a*_*l*_ is defined by utilizing [${\text{w}}_{i}^{-}(a_{l})$, ${\text{w}}_{i}^{\text{+}}(a_{l})$] (*i* = 1,…, *L*) and [*u*^*−*^(*H*_*n*_)(*a*_*l*_), *u*^*+*^(*H*_*n*_)(*a*_*l*_)] (*n* = 1, ..., *N*), which are applied further for the comparison of potential best alternatives in *M*_*b*_.

**Definition 1.** Assuming [${\text{w}}_{i}^{-}(a_{l})$, ${\text{w}}_{i}^{\text{+}}(a_{l})$] and [${\text{w}}_{i}^{-}(a_{m})$, ${\text{w}}_{i}^{\text{+}}(a_{m})$] enabling *l ≠ m* and *a*_*l*_, *a*_*m*_∈*M*_*b*_ are derived by resolving optimization issues stated in Section 4.3, the definition of interval difference of [${\text{w}}_{i}^{-}(a_{l})$, ${\text{w}}_{i}^{\text{+}}(a_{l})$] to [${\text{w}}_{i}^{-}(a_{m})$, ${\text{w}}_{i}^{\text{+}}(a_{m})$] is given as $\Delta {\text{ID}}_{lm}^{w_{i}}=C_{w_{i}}(a_{l})-C_{w_{i}}(a_{m})$=$\frac{d([{\text{w}}_{i}^{-}(a_{l}),{\text{w}}_{i}^{\text{+}}(a_{l})],[0,0])}{d([{\text{w}}_{i}^{-}(a_{l}),{\text{w}}_{i}^{\text{+}}(a_{l})],[0,0])+d([{\text{w}}_{i}^{-}(a_{l}),{\text{w}}_{i}^{\text{+}}(a_{l})],[1,1])}$$-\frac{d([{\text{w}}_{i}^{-}(a_{m}),{\text{w}}_{i}^{\text{+}}(a_{m})],[0,0])}{d([{\text{w}}_{i}^{-}(a_{m}),{\text{w}}_{i}^{\text{+}}(a_{m})],[0,0])+d([{\text{w}}_{i}^{-}(a_{m}),{\text{w}}_{i}^{\text{+}}(a_{m})],[1,1])}$.

Here, *d*([${\text{w}}_{i}^{-}(a_{l})$, ${\text{w}}_{i}^{\text{+}}(a_{l})$], [0,0]) and d([${\text{w}}_{i}^{-}(a_{l})$, ${\text{w}}_{i}^{\text{+}}(a_{l})$], [1,1]) represent the distance between [${\text{w}}_{i}^{-}(a_{l})$, ${\text{w}}_{i}^{\text{+}}(a_{l})$] and [0,0] and that between [${\text{w}}_{i}^{-}(a_{l})$, ${\text{w}}_{i}^{\text{+}}(a_{l})$] and [1,1], respectively. *d*([${\text{w}}_{i}^{-}(a_{m})$, ${\text{w}}_{i}^{\text{+}}(a_{\text{m}})$], [0,0]) and d([${\text{w}}_{i}^{-}(a_{m})$, ${\text{w}}_{i}^{\text{+}}(a_{\text{m}})$], [1,1]) stand severally for the distance from [${\text{w}}_{i}^{-}(a_{\text{m}})$, ${\text{w}}_{i}^{\text{+}}(a_{\text{m}})$] to [0,0] and that from [${\text{w}}_{i}^{-}(a_{\text{m}})$, ${\text{w}}_{i}^{\text{+}}(a_{\text{m}})$] to [1,1]. Their computation is accomplished using the distance measure between interval numbers by Li et al. (2008) [43], as presented in Definition A.2 in Appendix A.

When the interval difference of [${\text{w}}_{i}^{-}(a_{l})$, ${\text{w}}_{i}^{\text{+}}(a_{l})$] to [${\text{w}}_{i}^{-}(a_{m})$, ${\text{w}}_{i}^{\text{+}}(a_{m})$] is obtained as $\Delta {\text{ID}}_{lm}^{w_{i}}$, definition of interval difference for an alternative *a*_*l*_ in *M*_*b*_ under scenario two can be given by $\Delta {\text{ID}}_{lm}^{w_{i}}$ (*i* = 1, ..., L).

**Definition 2.** Assuming the interval difference of [${\text{w}}_{i}^{-}(a_{l})$, ${\text{w}}_{i}^{\text{+}}(a_{l})$] to [${\text{w}}_{i}^{-}(a_{m})$, ${\text{w}}_{i}^{\text{+}}(a_{m})$] is obtained as $\Delta {\text{ID}}_{lm}^{w_{i}}$, which enables *l ≠ m* and *a*_*l*_, *a*_*m*_∈*M*_*b*_, the differences in interval of alternatives *a*_*l*_ to *a*_*m*_ and of alternative *a*_*l*_ are formulated as


$$\begin{array}{c} \Delta ID_{lm}^{w}\text{=}\frac{\sum {\mathrm{\backslash nolimits}}_{i=1}^{L}(\Delta ID_{lm}^{w_{i}}-(-1))/2}{L} \end{array}$$ (44)

and


$$\begin{array}{c} \Delta {ID}_{l}^{w}\text{=}\frac{\sum {\mathrm{\backslash nolimits}}_{m=1,l\neq m}^{\left|M_{b}\right|}\Delta {ID}_{lm}^{w}}{\left|M_{b}\right|-1}, \end{array}$$ (45)

respectively.

Similarly, for an alternative al in Mb under scenario three, the interval difference is expressible with the utilization of [*u*^*−*^(*H*_*n*_)(*a*_*l*_), *u*^*+*^(*H*_*n*_)(*a*_*l*_)] (*n* = 1, ..., *N*).

**Definition 3.** Assuming [*u*^*−*^(*H*_*n*_)(*a*_*l*_), *u*^*+*^(*H*_*n*_)(*a*_*l*_)] and [*u*^*−*^(*H*_*n*_)(*a*_*m*_), *u*^*+*^(*H*_*n*_)(*a*_*m*_)] that enable *l ≠ m* and *a*_*l*_, *a*_*m*_∈*M*_*b*_ are derivable by solving optimization issues described in Section 4.3, the definition of interval difference of [*u*^*−*^(*H*_*n*_)(*a*_*l*_), *u*^*+*^(*H*_*n*_)(*a*_*l*_)] to [*u*^*−*^(*H*_*n*_)(*a*_*m*_), *u*^*+*^(*H*_*n*_)(*a*_*m*_)] is given as $\Delta {\text{ID}}_{lm}^{\text{u}(H_{n})}=C_{\text{u}(H_{n})}(a_{l})-C_{\text{u}(H_{n})}(a_{m})$= $\frac{d([u^{-}(H_{n})(a_{l}),u^{+}(H_{n})(a_{l})],[0,0])}{d([u^{-}(H_{n})(a_{l}),u^{+}(H_{n})(a_{l})],[0,0])+d([u^{-}(H_{n})(a_{l}),u^{+}(H_{n})(a_{l})],[1,1])}$$-\frac{d([u^{-}(H_{n})(a_{\text{m}}),u^{+}(H_{n})(a_{m})],[0,0])}{d([u^{-}(H_{n})(a_{\text{m}}),u^{+}(H_{n})(a_{m})],[0,0])+d([u^{-}(H_{n})(a_{\text{m}}),u^{+}(H_{n})(a_{m})],[1,1])}$.

Because [*u*^*−*^(*H*_1_)(*a*_*l*_), *u*^*+*^(*H*_1_)(*a*_*l*_)] =[0, 0] and [*u*^*−*^(*H*_*N*_)(*a*_*l*_), *u*^*+*^(*H*_*N*_)(*a*_*l*_)] =[1, 1], only [*u*^*−*^(*H*_*n*_)(*a*_*l*_), *u*^*+*^(*H*_*n*_)(*a*_*l*_)] (*n* = 2,..., *N* ˗ 1) are utilized in Definition 3.

When the interval difference of [*u*^*−*^(*H*_*n*_)(*a*_*l*_), *u*^*+*^(*H*_*n*_)(*a*_*l*_)] from [*u*^*−*^(*H*_*n*_)(*a*_*m*_), *u*^*+*^(*H*_*n*_)(*a*_*m*_)] is derived as $\Delta {\text{ID}}_{lm}^{u(H_{n})}$, for an alternative *a*_*l*_ in *M*_*b*_ under scenario two, definition of its interval difference can be given by $\Delta {\text{ID}}_{lm}^{\text{u}(H_{n})}$ (*n* = 2, ..., *N* ˗ 1).

**Definition 4.** Suppose that the interval difference of [*u*^*−*^(*H*_*n*_)(*a*_*l*_), *u*^*+*^(*H*_*n*_)(*a*_*l*_)] from [*u*^*−*^(*H*_*n*_)(*a*_*m*_), *u*^*+*^(*H*_*n*_)(*a*_*m*_)] is derived as $\Delta {\text{ID}}_{lm}^{\text{u}(H_{n})}$, which enables *l ≠ m* and *a*_*l*_, *a*_*m*_∈*M*_*b*_, the interval differences of alternatives *a*_*l*_ to *a*_*m*_ and of alternative *a*_*l*_ are expressable as


$$\begin{array}{c} \Delta ID_{lm}^{\text{u}}\text{=}\frac{\sum {\mathrm{\backslash nolimits}}_{n=2}^{N-1}(\Delta ID_{lm}^{u(H_{n})}-(-1))/2}{N-2} \end{array}$$ (46)

and


$$\begin{array}{c} \Delta ID_{l}^{\text{u}}\text{=}\frac{\sum {\mathrm{\backslash nolimits}}_{m=1,l\neq m}^{\left|M_{b}\right|}\Delta ID_{lm}^{u}}{\left|M_{b}\right|-1}, \end{array}$$ (47)

respectively.

The $\Delta {\text{ID}}_{lm}^{w_{i}}$, $\Delta {\text{ID}}_{lm}^{u(H_{n})}$, $\Delta {\text{ID}}_{lm}^{w}$, $\Delta {\text{ID}}_{lm}^{\text{u}}$, $\Delta {\text{ID}}_{l}^{w}$ and $\Delta {\text{ID}}_{l}^{\text{u}}$have the properties as follows.

**Property 1.** For $\Delta {\text{ID}}_{lm}^{w_{i}}$, $\Delta {\text{ID}}_{lm}^{u(H_{n})}$, $\Delta {\text{ID}}_{lm}^{w}$, $\Delta {\text{ID}}_{lm}^{\text{u}}$, $\Delta {\text{ID}}_{l}^{w}$ and $\Delta {\text{ID}}_{l}^{\text{u}}$ defined in Definitions 1−4, it is satisfied that

(1) $\Delta {\text{ID}}_{lm}^{w_{i}}\in [-1,1]$ and $\Delta {\text{ID}}_{lm}^{\text{u}(H_{n})}\in [-1,1]$,
(2) $\Delta {\text{ID}}_{lm}^{w_{\text{i}}}\text{=-}\Delta {\text{ID}}_{\text{ml}}^{w_{i}}$ and $\Delta {\text{ID}}_{lm}^{u(H_{n})}\text{=-}\Delta {\text{ID}}_{\text{ml}}^{u(H_{n})}$,
(3) $\Delta {\text{ID}}_{lm}^{w}\in [0,1]$ and $\Delta {\text{ID}}_{lm}^{\text{u}}\in [0,1]$, and
(4) $\Delta {\text{ID}}_{l}^{w}\in [0,1]$ and $\Delta {\text{ID}}_{l}^{\text{u}}\in [0,1]$.

On the bases of Definitions 1−4, the difference in interval for the optional al in Mb under three scenarios is formulated as


$$\Delta ID_{l}^{w,u}\;=\;\left(\Delta ID_{l}^{w}+\Delta ID_{l}^{u}\right)/2$$ (48)

Because $\Delta {\text{ID}}_{l}^{w}\in [0,1]$ and $\Delta {\text{ID}}_{l}^{\text{u}}\in [0,1]$, the scope of$\Delta {\text{ID}}_{l}^{w,\text{u}}$is within [0,1]. In *M*_*b*_, an alternative *a*_*l*_ with greater difference in interval is favored over other alternatives. Thus, generation of alternative ranking order is achievable with the exploitation of interval difference between potentially best alternatives in *M*_*b*_.

For MADA problems, we usually only need to deal with one of three situations. Through several iterations, we can identify and compare the potential optimal solutions, and then produce a complete solution ranking, as shown below.

### 4.5. Procedure of the proposed method

**Fig 2:**
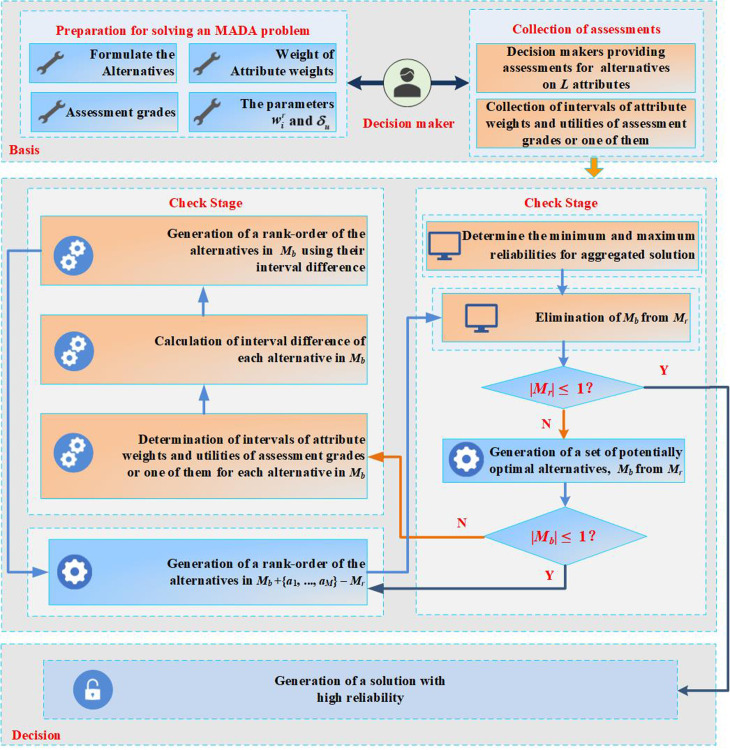
A flow diagram for our approach.

Taking into account what has been introduced in Sections 4.1–4.4, the solution seeking process in which the aggregated solution reliability is incorporated is depicted in Fig 2.

**Step 1:** Construct a multi−attribute decision problem
*L* attributes along with respective classes (benefits or costs) are identified by decision maker, as well as *N* evaluation levels. Further, a multi−attribute decision problem is established by listing *M* alternatives.
**Step 2:** To tackle the established decision problem, an ER method exploiting high solution reliability is put forward.
One scenario is specified by decision maker among three scenarios. Under scenarios one and three, the decision maker provides *δ*_*u*_. Under scenarios two and three, *u*(*H*_*n*_) (*n* = 1, ..., *N*) are provided, which enable 0 = *u*(*H*_1_) < *u*(*H*_2_) < < *u*(*H*_*N*_) = 1 and the attribute weight set *w*. In addition, assuming *M*_*b*_ = {Ø} and *M*_*r*_ = {*a*_1_, ..., *a*_*M*_}.
**Step 3:** Gather evaluation values for the alternatives.
The decision maker provides an estimate of the solution on every attribute.
**Step 4:** Gather the interval values for attribute weights and/or evaluate the assessment grades utility.
In a particular case, an interval of attribute weights and/or the assessment grade utility is/are provided by decision maker.
**Step 5:** Determine the minimum and maximum reliabilities for aggregated solution.
Minimum and maximum aggregated solution reliabilities for the alternative *a*_*l*_, i.e. *Q*^−^(*a*_*l*_) and *Q*^*+*^(*a*_*l*_), are determined by tackling nonlinear optimization problems expressed in Eqs. (19)–(20).
**Step 6:** Exclude *M*_*b*_ from *M*_*r*_.
In the present iteration, collection *M*_*b*_ is excluded from *M*_*r*_.
**Step 7:** Determine if |*M*_*r*_| falls below or is equivalent to 1.
In case |*M*_*r*_| falls below or is equivalent to 1, the complete order is derived for *M* alternatives, followed by proceeding to step 13; Otherwise, enter step 8.
**Step 8:** Identify *M*_*b*_ from *M*_*r*_.
In a specific case, the set *M*_*b*_ under the present iteration is identified based on alternatives set *M*_*r*_, according to the optimal solution of the optimization model in Eqs. (8)–(18).
**Step 9:** Determine if |*M*_*b*_| is equivalent to 1.
In case |*M*_*b*_| is equivalent to 1, proceed to step 13. Otherwise, enter step 10.
**Step 10:** Determine optimal attribute weight interval or evaluate the utility of the level.
In the current iteration, for the scheme from the set *M*_*b*_, the utility of the optimal interval or evaluation level of its attribute weights is derived by addressing the pairwise optimization model under a particular circumstance.
**Step 11:** Calculate interval deviation for every scheme in *M*_*b*_.
To tackle the multi−attribute decision problem in a specific case, computation of interval deviation is accomplished for every alternative in *M*_*b*_ under the present iteration.
**Step 12:** Derive a rank−order for alternatives in the collection *M*_*b*_ + {*a*_*1*_,..., *a*_*M*_}− *M*_*r*_.
In case |*Mb*| is equivalent to 1, the current iteration directly produces alternatives order in the set *M*_*b*_ + {*a*_*1*_,..., *a*_*M*_} − *M*_*r*_. Otherwise, inter−alternative interval differences in the present iteration Mb are used to produce alternative order, which in addition can be blended with the order obtained in the prior iteration, and then produced alternative sorting in *M*_*b*_ + {*a*_*1*_,..., *a*_*M*_} − *M*_*r*_. Thereafter, return to step 5.
**Step 13:** Complete the process.
Finally, the order of *M* alternative can be obtained, i.e., the solution of multi−attribute decision problem.

## 5. Case study

In the present section, applicability and effectiveness of our proposed approach are confirmed by analyzing a car performance assessment (CPA) problem, where the entire optimization problems are developed on the MATLAB platform. Sections below elaborate on the above CPA problem in detail, and solve it under three settings for comparison of relevant solutions and iterations.

### 5.1. Description of the CPA problem

CPA issue assesses six commercial vehicles *c*_*l*_ (*l* =1, …, 6) regarding seven performance attributes, namely acceleration, handling, braking, power transmission, fuel economy, horsepower and ride mass. Assuming the assessment grades are denoted as *Ω =* {*H*_*n*_, *n*=1, …, 6} = {Worst, Poor, Average, Good, Excellent, Top} = {*W*, *P*, *A*, *G*, *E*, *T*}. With the utilization of assessment grades, the six vehicles are evaluated by decision maker, and Table 3 details relevant results, which comprise complete ignorance assessments (*β*_*Ω*,*i*_(*a*_*l*_) = 1) and incomplete assessments (*β*_*Ω*,*i*_(*a*_*l*_) > 0). The description for the CPA dataset is given in Appendix B. Decision maker wants to get their ranking order, a CPA issue solution, through performance contrast among six cars. CPA issues in these three cases are addressed to illustrate the flexibility of our proposed method. Decision maker provides relevant parameters for these three scenarios below.

**Table 3:** Assessments for six executive vehicles in CPA issue.

Performance	*c*_1_	*c*_2_	*c*_3_	*c*_4_	*c*_5_	*c*_6_
Acceleration (e1)	{(P,0.2), (A,0.8))}	{(G,0.5), (E,0.5)}	{(G,0.5), (E,0.5)}	{(A,0.4), (G,0.6)}	{(G,0.5), (E,0.5)}	{(G,0.5), (E,0.5)}
Braking (e2)	{(G,1.0)}	{(E,0.333), (T,0.667)}	{(G,0.5), (E,0.5)}	{(P,0.75), (A,0.25)}	{(P,1.0)}	{(E,1.0)}
Handling (e3)	{(A,0.4), (G,0.6)}	{(E,0.6), (T,0.4)}	{(A,0.4), (G,0.4), (Ω,0.2)}	{(A,1.0)}	{(G,1.0)}	{(E,0.5), (T,0.4), (Ω,0.1)}
Horsepower (e4)	{(E,0.333), (T,0.667)}	{(P,0.533), (A,0.467)}	{(G,0.462), (E,0.538)}	{(G,0.385), (E,0.615)}	{(W,0.467), (P,0.533)}	{(A,0.267), (G,0.733)}
Ride quality (e5)	{(G,0.6), (E,0.4)}	{(A,1.0)}	{(Ω,1)}	{(G,1.0)}	{(G,1.0)}	{(Ω,1)}
Power train (e6)	{(A,0.4), (G,0.6)}	{(G,1.0)}	{(E,0.5), (T,0.4), (Ω,0.1)}	{(A,0.4), (G,0.6)}	{(G,0.6), (E,0.4)}	{(E,0.4), (T,0.4), (Ω,0.2)}
Fuel economy (e7)	{(G,1.0)}	{(G,1.0)}	{(E,1.0)}	{(G,1.0)}	{(A,1.0)}	{(G,1.0)}

Scenario one: Assuming that ${\text{w}}_{i}^{\text{r}}$ (*i* = 1,. . .,7) = (0.01, 0.01,0.01,0.01,0.01,0.01,0.01), [${\text{w}}_{i}^{-}$, ${\text{w}}_{i}^{\text{+}}$](*i* = 1,. . .,7) = {[0.05,0.45], [0.05,0.45], [0.05,0.45], [0.05,0.45], [0.05,0.45], [0.05,0.45], [0.05,0.45]}, and [*u*^*−*^(*H*_*n*_), *u*^*+*^(*H*_*n*_)] (*n* = 1,. . .,6) = {[0,0], [0.1,0.3], [0.3,0.5], [0.5,0.7], [0.7,0.9], [1,1]}.

Scenario two: Assuming that *u*(*H*_*n*_) (*n* = 1,. . .,6) = (0, 0.2, 0.4, 0.6, 0.8, 1), ${\text{w}}_{i}^{\text{r}}$ (*i* = 1,. . .,7) = (0.01, 0.01, 0.01, 0.01, 0.01, 0.01, 0.01), and [${\text{w}}_{i}^{-}$, ${\text{w}}_{i}^{\text{+}}$](*i* = 1,. . .,7)= {[0.1,0.35], [0.1,0.35], [0.1, 0.35], [0.1,0.35], [0.1,0.35], [0.1,0.35], [0.1,0.35]}.

Scenario three: Assuming *w* = (1/7,1/7,1/7,1/7,1/7,1/7,1/7) and [*u*^*−*^(*H*_*n*_), *u*^*+*^(*H*_*n*_)] (*n* = 1, ..., 6) = {[0,0], [0.1,0.3], [0.3,0.5], [0.5,0.7], [0.7,0.9], [1,1]}. Under scenarios one and three, *δ*_*u*_ = 0.05 is specified by decision maker. The first step is designation of *M*_*b*_ = {Ø} and *M*_*r*_ = {*c*_1_, ..., *c*_6_}. Null collection is symbolized by ‘Ø’.

### 5.2. Generation of a solution to the CPA problem in the first scenario

Under scenario one, optimization model in Eqs. (8)–(18) is addressed with specified ${\text{w}}_{i}^{\text{r}}$, [${\text{w}}_{i}^{-}$, ${\text{w}}_{i}^{\text{+}}$](*i* = 1,. . .,7), [*u*^*−*^(*H*_*n*_), *u*^*+*^(*H*_*n*_)], and *δ*_*u*_, thereby obtaining the set *M*_*b*_ = {*c*_1_, *c*_2_, *c*_3_, *c*_5_, *c*_6_} from optimal solutions. It be can then derived that *M*_*r*_ = {*c*_4_} during iteration one. Since |*M*_*b*_| > 1, computation of interval difference should be accomplished for five vehicles in *M*_*b*_ for their comparison purpose. Then, five sets of [${\text{w}}_{i}^{-}$(*c*_*l*_), ${\text{w}}_{i}^{\text{+}}$(*c*_*l*_)] (*i* = 1, ..., 7) and [*u*^*−*^(*H*_*n*_)(*c*_*l*_), *u*^*+*^(*H*_*n*_)(*c*_*l*_)] are calculated through resolution of the optimization problem pair in Eqs. (21)–(30). Table 4 details relevant results, based on which we calculate that $\Delta {\text{ID}}_{l}^{w}$ = {0.5453,0.5906,0.5602,0.2134,0.5894} and $\Delta {\text{ID}}_{l}^{\text{u}}$ = {0.5341, 0.5341,0.5341,0.3421,0.5341} using Definitions 1-4.

**Table 4:** Attribute weights of five vehicles in Mb during iteration one under scenario one, and the optimization interval for evaluating the rating utility.

The cars in *M*_*b*_	[${\text{w}}_{i}^{-}$(*c*_*l*_), ${\text{w}}_{i}^{\text{+}}$(*c*_*l*_)] (*i* = 1, ..., 7) and [*u*^*−*^(*H*_*n*_)(*c*_*l*_), *u*^*+*^(*H*_*n*_)(*c*_*l*_)] (*n* = 1, …, 6)
*c*_1_	{[0.05,0.2879], [0.05,0.4017], [0.05,0.2885], [0.05,0.45], [0.05,0.45], [0.05,0.3855], [0.05,0.3994]}, {[0,0], [0.1,0.3], [0.3,0.5], [0.5,0.7], [0.7,0.9], [1,1]}
*c*_2_	{[0.05,0.45], [0.05,0.45], [0.05,0.45], [0.05,0.2773], [0.05,0.3900], [0.05,0.45], [0.05,0.45]}, {[0,0], [0.1,0.3], [0.3,0.5], [0.5,0.7], [0.7,0.9], [1,1]}
*c*_3_	{[0.05,0.45], [0.05,0.3217], [0.05,0.2785], [0.05,0.45], [0.05,0.3188], [0.05,0.45], [0.05,0.45]}, {[0,0], [0.1,0.3], [0.3,0.5], [0.5,0.7], [0.7,0.9], [1,1]}
*c*_5_	{[0.25,0.3496], [0.05,0.1338], [0.05,0.2431], [0.05,0.1253], [0.24,0.37], [0.05,0.3678], [0.05,0.147]}, {[0,0], [0.2465,0.2891], [0.3,0.3007], [0.5,0.7], [0.7,0.9], [1,1]}
*c*_6_	{[0.05,0.45], [0.05,0.45], [0.05,0.45], [0.05,0.2677], [0.05,0.3751], [0.05,0.45], [0.05,0.3567]}, {[0,0], [0.1,0.3], [0.3,0.5], [0.5,0.7], [0.7,0.9], [1,1]}

Base on $\Delta {\text{ID}}_{l}^{w}$ and $\Delta {\text{ID}}_{l}^{\text{u}}$, we have $\Delta {\text{ID}}_{l}^{w,\text{u}}$= ($\Delta {\text{ID}}_{l}^{w}$+$\Delta {\text{ID}}_{l}^{\text{u}}$)/2 = {0.5397, 0.5624, 0.5472, 0.2778, 0.5617}. We then obtain a rank order for five vehicles in *M*_*b*_ by exploiting $\Delta {\text{ID}}_{l}^{w,\text{u}}$as *c*_2_ > *c*_6_ > *c*_3_ *c*_1_ > *c*_5_. Since |*M*_*r*_| = 1 during iteration one, a complete ranking order for six vehicles is infer that *c*_2_ > *c*_6_ > *c*_3_ > *c*_1_ > *c*_5_ > *c*_4_. We deem it as the CPA issue solution under scenario one. As is clear from the ranking order for six vehicles, *c*_2_ is the best-performing vehicle.

For a more universal situation, assuming that ${\text{w}}_{i}^{+}$ (*i* = 1, ..., 7) = (1,1,1,1,1,1,1) and *u*^+^(*H*_*n*_) (*n* = 2, ..., 5) = (1, 1, 1, 1). The set *M*_*b*_ and *M*_*r*_ are respectively obtained as {*c*_1_, *c*_2_, *c*_3_, *c*_4_, *c*_5_, *c*_6_} and {Ø} during iteration one. We then obtain that $\Delta {\text{ID}}_{l}^{w}$ = {0.5399, 0.5487, 0.5346, 0.448, 0.3597, 0.5609}, $\Delta {\text{ID}}_{l}^{\text{u}}$ = {0.5, 0.5, 0.5, 0.5, 0.5, 0.5}, and $\Delta {\text{ID}}_{l}^{w,\text{u}}$ = {0.52, 0.5244, 0.5173, 0.474, 0.4299, 0.5305} for six vehicles in *M*_*b*_. Base on $\Delta {\text{ID}}_{l}^{w,\text{u}}$, a thorough ranking order for six vehicles is infer that *c*_6_ > *c*_2_ > *c*_1_ > *c*_3_ > *c*_4_ > *c*_5_, which is considered as the CPA issue solution. The solution and highest vehicle performance have changed significantly.

### 5.3. Generation of a solution to the CPA problem in the second scenario

Under scenario two, optimization model in Eqs. (8)–(18) is addressed using exact *u*(*H*_*n*_) (*n* = 1,. . .,6), ${\text{w}}_{i}^{\text{r}}$ (*i* = 1,. . .,7), and [${\text{w}}_{i}^{-}$, ${\text{w}}_{i}^{\text{+}}$] (*i* = 1,. . .,7), thereby obtaining a set *M*_*b*_ = {*c*_1_, *c*_2_, *c*_3_, *c*_6_}. It is clear from set *M*_*b*_ that *M*_*r*_ = {*c*_4_, *c*_5_} during iteration one. Since |*M*_*b*_| > 1, computation of interval difference should be accomplished for four vehicles in *M*_*b*_ for comparison among these cars. To achieve this, the optimization problem pair in Eqs. (31)–(36) for four vehicles in *M*_*b*_ during iteration one are solved to derive four sets of [${\text{w}}_{i}^{-}$(*c*_*l*_), ${\text{w}}_{i}^{\text{+}}$(*c*_*l*_)] (*i* = 1,. . . ,7), which are shown in Table 5. Then, based on Definitions 1 and 2, $\Delta {\text{ID}}_{l}^{w}$ enabling *c*_*l*_ ∈*M*_*b*_ is computed in terms of Table 5 outcomes as {0.4651,0.5237,0.502,0.507} during such iteration. A rank-order of four vehicles in *M*_*b*_ is produced as *c*_2_ > *c*_6_ > *c*_3_ > *c*_1_. Since |*M*_*r*_| > 1, iteration should be carried out. The optimization model in Eqs. (8)–(18) is addressed, thereby getting *M*_*b*_ = {*c*_4_, *c*_5_} during iteration two. It can be obtained the *M*_*r*_ = {Ø} during such iteration. By tackling the optimization problems pair in Eqs. (31)–(36), the [${\text{w}}_{i}^{-}$(*c*_*l*_), ${\text{w}}_{i}^{\text{+}}$(*c*_*l*_)] (*l* = 4,5) are calculated as {[0.1,0.35], [0.1,0.35], [0.1,0.35], [0.1,0.35], [0.1,0.35], [0.1,0.35], [0.1,0.35]} and {[0.1,0.35], [0.1,0.15], [0.1,0.35], [0.1,0.12], [0.1,0.35], [0.1,0.35], [0.1,0.135]}, respectively. It has $\Delta {\text{ID}}_{4}^{w}$ > $\Delta {\text{ID}}_{5}^{w}$ in terms of Definitions 1 and 2. Therefore, an intact ranking order for six vehicles is inferred as *c*_2_ > *c*_6_ > *c*_3_ > *c*_1_ > *c*_4_ > *c*_5_.

**Table 5:** In the second case, the optimization interval of the attribute weights for four vehicles in Mb during iteration one.

Vehicles in *M*_*b*_	[${\text{w}}_{i}^{-}$(*c*_*l*_), ${\text{w}}_{i}^{\text{+}}$(*c*_*l*_)] (*i* = 1, …, 7)
*c*_1_	{[0.1,0.2231], [0.1,0.1874], [0.1,0.2531], [0.1,0.35], [0.1,0.35], [0.1,0.1865], [0.1,0.2541]}
*c*_2_	{[0.1,0.35], [0.1,0.35], [0.1,0.2551], [0.1,0.19], [0.1,0.35], [0.1,0.2288], [0.1,0.2345]}
*c*_3_	{[0.1,0.35], [0.1,0.2487], [0.1,0.1689], [0.1,0.35], [0.1,0.2167], [0.1,0.35], [0.1,0.35]}
*c*_6_	{[0.1,0.35], [0.1,0.35], [0.1,0.35], [0.1,0.2245], [0.1,0.1798], [0.1,0.35], [0.1,0.2342]}

Resembling scenario one, assuming ${\text{w}}_{i}^{\text{+}}$ (*i* = 1, ..., 7) = (1,1,1,1,1,1,1), *M*_*b*_ and *M*_*r*_ during iteration one are then calculated as {*c*_1_, *c*_2_, *c*_3_, *c*_5_, *c*_6_} and {*c*_4_}, respectively. Then, $\Delta {\text{ID}}_{\text{l}}^{\text{w}}$ = {0.4875, 0.6231,0.5231,0.3647,0.5501} is obtained for five vehicles in set *M*_*b*_. Since |*M*_*r*_| = 1 during iteration one, an intact rank order for six vehicles is derivable as *c*_2_ > *c*_6_ > *c*_3_ > *c*_1_ > *c*_5_ > *c*_4_, representing the CPA issue solution. Based on the result, *c*_2_ remains to be the best-performing vehicle although the solution is changed.

### 5.4. Generation of a solution to the CPA problem in the third scenario

Under scenario three, optimization model in Eqs. (8)–(18) is addressed using exact *w*, [*u*^*−*^(*H*_*n*_), *u*^* + *^(*H*_*n*_)] (*n* =  1,...,6), and *δ*_*u*_, thereby calculating set *M*_*b*_ as {*c*_1_, *c*_2_, *c*_3_, *c*_6_} during iteration one. The set *M*_*r*_ is inferred as {*c*_4_, *c*_5_}. Since | *M*_*b*_ | >  1, interval difference computation should be accomplished for four vehicles in *M*_*b*_ to allow their comparison. By tackling the optimization problems pair in Eqs. (37)–(43), four sets of [*u*^*−*^(*H*_*n*_)(*c*_*l*_), *u*^* + *^(*H*_*n*_)(*c*_*l*_)] (*n* =  1,...,6) are obtained during iteration one, as listed in Table 6. Based on the table outcomes, Definitions 3 and 4 are exploited to derive $\Delta {\text{ID}}_{l}^{\text{u}}$ = {0.1674, 0.6772, 0.5766, 0.6145} for four vehicles in *M*_*b*_ during iteration one. Subsequent step is creation of a rank-order for these four vehicles, that is, *c*_2_ > *c*_6_ > *c*_3_ > *c*_1_. Since | *M*_*r*_ | >  1, iteration should be carried out. Optimization model in Eqs. (8)–(18) is addressed for *c*_4_ and *c*_5_, thereby determining *M*_*b*_ =  {*c*_4_} during iteration two from best solutions, which means that the corresponding *M*_*r*_ =  {*c*_5_}. Therefore, the complete ranking order is generated as *c*_2_ > *c*_6_ > *c*_3_ > *c*_1_ > *c*_4_ > *c*_5_ for the six vehicles. Resembling the first scenario, assuming *u*^* + *^(*H*_*n*_) (*n* =  2,..., 5) =  (1,1,1,1), *M*_*b*_ and *M*_*r*_ during iteration one are calculated separately as {*c*_1_, *c*_2_, *c*_3_, *c*_6_} and {*c*_4_, *c*_5_}. For the four cars in the set *M*_*b*_, the $\Delta {\text{ID}}_{l}^{\text{w}}$ is recalculated as {0.3763,0.6456,0.499,0.5218}, leading to creation of their ranking order, i.e., *c*_2_ > *c*_6_ > *c*_3_ > *c*_1_. This finishes iteration one. Since | *M*_*r*_ | >  1, it is necessary to carry out iteration two, during which *M*_*b*_ =  {*c*_4_, *c*_5_} is determined by seeking best solutions for the optimization model in Eqs. (8)–(18). Next step is determination of corresponding *M*_*r*_ as {Ø}. Then, through resolution of optimization problem pair in Eqs. (37)–(43) for *c*_4_ and *c*_5_, it can be generated that [*u*^*−*^(*H*_*n*_)(*c*_*l*_), *u*^* + *^(*H*_*n*_)(*c*_*l*_)] (*n* =  1,..., 6, *l* =  4,5) =  {[0], [0.04,0.7], [0.15,0.75], [0.25,0.85], [0.25,0.9], [1]}, {[0], [0.05,0.2784], [0.15,0.334], [0.45,0.498], [0.453,0.95], [1]}. This infers that *c*_4_ > *c*_5_ by Definitions 3 and 4. Hence, an intact ranking order is derivable as *c*_2_ > *c*_6_ > *c*_3_ > *c*_1_ > *c*_4_ > *c*_5_ for all six vehicles, without changing the solution.

**Table 6:** In the third case, optimization intervals of evaluation level for four vehicles in *M*_*b*_ during iteration one.

Vehicles in *M*_*b*_	[*u*^*−*^(*H*_*n*_)(*c*_l_), *u*^*+*^(*H*_*n*_)(*c*_l_)] (*n* = 1,..., 6)
*c*_1_	{[0,0], [0.1,0.1221], [0.4,0.5], [0.65,0.7], [0.74,0.75], [1]}
*c*_2_	{[0,0], [0.1,0.3], [0.31,0.5], [0.5,0.7], [0.7,0.9], [1]}
*c*_3_	{[0,0], [0.1,0.3], [0.3,0.5], [0.5,0.7], [0.75,0.9], [1]}
*c*_6_	{[0,0], [0.1,0.3], [0.3,0.45], [0.5,0.7], [0.7,0.9], [1]}

In conclusion, under the parameters provided in Section 5.1, the solution to the CPA problem in these three cases is very similar apart from the varying order of *c*_4_ and *c*_5_. In the solution, *c*_2_ consistently has the best performance. Nonetheless, solution for scenario one is found by single iteration, while that for scenarios two and three are found by double iterations. Specifically, during the second iteration, *c*_4_ is contrasted against *c*_5_ by the interval difference in the second situations, while in the third situation, we distinguish them directly due to *M*_*b*_ =  {*c*_4_}. Additionally, when ${\text{w}}_{i}^{\text{+}}$ (*i* =  1,..., 7) along with *u*^* + *^(*H*_*n*_) (*n* =  2,..., 5) are given, the best−performing car is probably replaced, as under the first scenario.

### 5.5. Comparison analysis

To compare the objective derivation of attribute weights herein with that in representative references, we describe the way of objectively identifying attribute weights based on a belief decision matrix by following original processes of entropy [32], CRITIC [33], SD [33], as well as CCSD [27], the detail processes of the four typical methods are presented in Appendix C.

To contrast our proposed method against these four methods, estimation of attribute weights and production of CPA issue solutions are accomplished with four extended models under requisites prescribed in Section 5.1. Get the attribute weight [*u*_min_(*c*_*l*_), *u*_max_(*c*_*l*_)] (*l* =  1,..., 6), as well as rank-order of *c*_*l*_ (*l* =  1,..., 6). Table 7 details the computational outcomes.

**Table 7:** Attribute weights, [*u*_min_(*c*_*l*_), *u*_max_(*c*_*l*_))] (*l* =  1,..., 6), Reliability along with rank-orders of *c*_*l*_ (*l* =  1,..., 6) derived using four approaches in CPA issue.

Methods	Attribute weights	[*u*_min_(*c*_*l*_), *u*_max_(*c*_*l*_))]	Reliability	Rank order
Entropy	(0.05, 0.1418, 0.0729, 0.1824, 0.4241, 0.05, 0.0788)	{[0.6284,0.6284], [0.4348,0.4348], [0.448,0.8312], [0.4872,0.4872], [0.4007,0.4007], [0.4142, 0.805]}	0.2645	*c*_1_ > *c*_4_ > *c*_3_ > *c*_2_ > *c*_6_ > *c*_5_
SD	(0.0946, 0.1898, 0.1192, 0.2074, 0.201,0.0619, 0.1261)	{[0.7161,0.7161], [0.6497,0.6497], [0.6571,0.8348], [0.599,0.599], [0.4637,0.4637], [0.6408,0.8214]}	0.365	*c*_1_ > *c*_3_ > *c*_2_ > *c*_6_ > *c*_4_ > *c*_5_
CRITIC	(0.0781, 0.1942, 0.1148, 0.2319, 0.2106, 0.05, 0.1205)	{[0.7282,0.7282], [0.6291,0.6291], [0.651,0.8331], [0.6019,0.6019], [0.4407,0.4407], [0.6286, 0.8139]}	0.3799	*c*_1_ > *c*_3_ > *c*_2_ > *c*_6_ > *c*_4_ > *c*_5_
CCSD	(0.0803, 0.1977, 0.1188, 0.2192, 0.2144, 0.05, 0.1196)	{[0.7231,0.7231], [0.638,0.638], [0.6459,0.8327], [0.597,0.597], [0.4486,0.4486], [0.6296,0.8191]}	0.3061	*c*_1_ > *c*_3_ > *c*_2_ > *c*_6_ > *c*_4_ > *c*_5_
Proposed method	——	——	0.4043	*c*_6_ > *c*_2_ > *c*_1_ > *c*_3_ > *c*_4_ > *c*_5_

As can be seen from Table 7, unlike *c*_3_ which is derived from proposed method, *c*_1_ is the optimal choice derived based on the four approaches. This is primarily attributable to failure to address the alternative insensitivities to the attribute weight alterations with these four approaches. The optimization model generalized by the four approaches involves *L* equation. Hence, an exclusive *w*_*i*_ (*i* =  1,..., *L*) set can be decided by every model, thus generating an exclusive solution. Contrastively, our proposed method addresses the solution insensitivity to the attribute weight alterations during generation of a high reliability solution for the CPA issue.

From the results in Table 7, the different reliability of the aggregated solutions is derived from the four representative methods and the proposed method. Compared with the proposed method, the low recognizable expected utilities are created using the four representative methods, which further lead to the low reliability of the aggregated solutions. Different from the four methods, the high recognizable interval-valued expected utilities are generated by using the proposed method, which contribute to the generation of highest reliability of the aggregated solutions. Due to the high solution reliability created by the proposed method, the preferential order of six cars seems more reliable and convincing than the other four methods.

When the methods of entropy, SD, CRITIC, and CCSD are used to evaluate the performance of the six vehicles, the most best-performing vehicle is *c*_1_. For the CPA problem, the decision maker attempts to take into account the worst scenarios of each vehicle. In accordance with the willingness of the decision maker, the reliability of each vehicle is considered. Under the conditions, the solution generated by the proposed method, i.e., *c*_1_,..., *c*_6_ with the preferential order of *c*_6_ > *c*_2_ > *c*_1_ > *c*_3_ > *c*_4_ > *c*_5_, is clearly different from those associated with the four methods. When the solution reliability is considered, the solution generated by the proposed method is also different from those associated with the four methods. The reason why the solution generated by the proposed method is considered as what the decision maker anticipates is that the DM is involved in the process of obtaining all possible sets of attribute weights when generating the expected utilities of the six cars. The proposed method to determine order reflects the interaction between the experts and decision maker making the obtained solution consistent with the real preferences of the decision maker. Relevant theoretical analysis can be found in Section 4.2. The reliability of the ranking order, however, is not considered in the obtainment of solution by using the four methods.

In brief, a method for objectively determining attribute weights is proposed with the consideration of solution reliability. The proposed method can help guarantee the generation of a solution with high reliability.

## 6. Numerical and Simulation Experiments analysis

In Section 5, it does not seem convincing enough to show the superiority of the proposed method by a single specific problem. In order to further prove the superiority of the proposed method, a numerical and simulation experiment analysis is designed in this section.

### 6.1. Numerical analysis

In this section, we conduct a numerical experiment to analyze the superiority of the proposed method. This numerical experiment is based on the practical example presented in Fu et al (2014) [42]. This numerical experiment investigates the selection of leading industries in the industry-cluster region in the north of the Yangtze River. The data related with the leading industries selection problem is presented in Table D.1 of Appendix D.

In this numerical experiment, following the steps outlined in Sect. 4.5, the reliability of the aggregated solutions and the rank-order of the twelve industries is calculated firstly using the assessment data in Table D.1, as presented in Table 8. The four methods in Appendix C are used to generate four solutions to the problem of selecting leading industries. The resulting reliability of the aggregated solutions are compared with those using the proposed method, as presented in Table 8.

**Table 8:** Comparison of rank-order and reliability of the aggregated solutions and generated by the five methods and proposed method.

Methods	Rank-order	*Q*
Entropy	*I*_9_ ≻ *I*_5_ ≻ *I*_3_ ≻ *I*_6_ ≻ *I*_2_ ≻ *I*_10_ ≻ *I*_11_ ≻ *I*_4_ ≻ *I*_7_ ≻ *I*_8_ ≻ *I*_12_ ≻ *I*_1_	1.241
SD	*I*_5_ ≻ *I*_9_ ≻ *I*_6_ ≻ *I*_2_ ≻ *I*_3_ ≻ *I*_4_ ≻ *I*_10_ ≻ *I*_11_ ≻ *I*_7_ ≻ *I*_8_ ≻ *I*_12_ ≻ *I*_1_	0.8334
CRITIC	*I*_5_ ≻ *I*_9_ ≻ *I*_6_ ≻ *I*_3_ ≻ *I*_2_ ≻ *I*_10_ ≻ *I*_4_ ≻ *I*_11_ ≻ *I*_7_ ≻ *I*_8_ ≻ *I*_12_ ≻ *I*_1_	0.909
CCSD	*I*_5_ ≻ *I*_9_ ≻ *I*_6_ ≻ *I*_3_ ≻ *I*_2_ ≻ *I*_10_ ≻ *I*_4_ ≻ *I*_11_ ≻ *I*_8_ ≻ *I*_7_ ≻ *I*_1_ ≻ *I*_12_	0.8063
Proposed Method	*I*_9_ ≻ *I*_5_ ≻ *I*_6_ ≻ *I*_2_ ≻ *I*_11_ ≻ *I*_7_ ≻ *I*_8_ ≻ *I*_12_ ≻ *I*_1_ ≻ *I*_3_ ≻ *I*_4_ ≻ *I*_10_	1.2362

It is shown from Table 4 that the rank-order of the industries generated by the five methods is correspondingly different although the top five industries obtained by the five methods are the same. In particular, in terms of the minimal satisfaction produced, the second and the third industries are very close if using SD, CRITIC, CCSD, and the proposed methods, while using the entropy method, it is the second and the sixth that are very close. This results in a clear difference between the rank-order of the second, the third, and the sixth industries generated by the entropy method and those by the other four methods. In brief, the proposed method can generate a reasonable set of attribute weights by using solution reliability and uses the attribute weights to guarantee high reliability of the aggregated solution.

### 6.2. Simulation experiments analysis

In the simulation experiment, six possible alternative and attribute combinations as shown in Table 9 were selected. In order to ensure the statistical significance of the simulation experiment, 100 random belief decision matrices were generated for each combination to conduct the simulation experiment. Then, with the changes of alternatives and attributes, simulation experiments are designed, as shown in Appendix D of Table D.2.

**Table 9:** The random decision matrices with different combinations of alternatives and attributes.

No	Number of alternatives (*L*)	Number of attributes (*L*)
1	5	5
2	6	6
3	7	7
4	8	8
5	9	9
6	10	10

**Fig 3:**
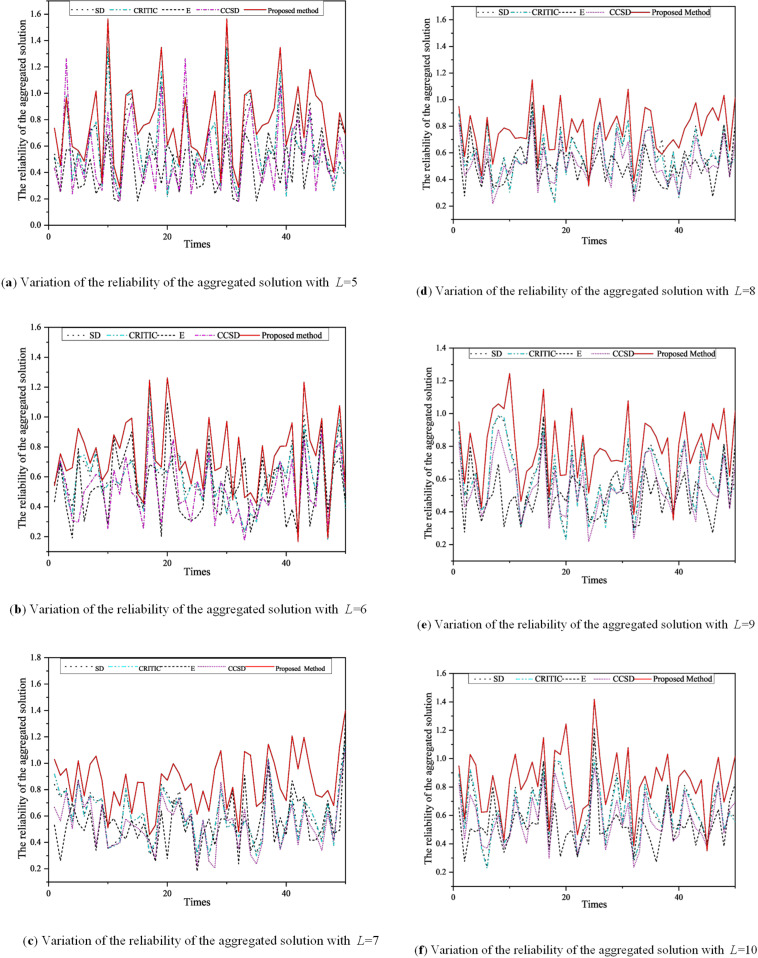
Variation of the reliability of the aggregated solution for the four representative methods and proposed method.

Based on the simulation process in Table D.1, 600 groups of randomized experiments were conducted to verify the superiority of the proposed method. From the results of simulation experiments, five different curves of the reliability of the aggregated solutions generated by the four representative methods and the proposed methods are shown in Fig 3.

As shown in Fig 3, with the increase of *L*, the reliability of the aggregated solution generated by the proposed method is always greater than that of the four representative methods. In addition, the reliability trend of the aggregated solutions generated by this method is consistent with that of the four representative methods. These results show that the proposed method is the most effective way to identify and measure the performance differences of alternative solutions on various attributes. In other words, the attribute weights generated using the methods presented in this paper may be more reasonable than the weights of the four representative methods. The above results in Fig 3 show that compared with the four representative methods, the proposed method is the best method to meet the requirements of solving high reliability.

On the other hand, in order to further prove the superiority of the proposed method, the significance of testing the superiority of the proposed method is investigated by the following statistical methods. From a statistical point of view, since the overall distribution of the results of 600 randomized experiments was unknown, this paper adopted non-parametric Wilcoxon rank sum test. Table 10 shows the basic content of Wilcoxon rank sum test. As shown in Table 10, when the significance level is 5%, it can be considered that the reliability of the polymerization solution generated by the method in this paper is significantly higher than that of other methods. The test results are consistent with the simulation results shown in Fig 3.

**Table 10:** Wilcoxon signed-rank test results at the significance level *α* =  0.05.

Cases	Null hypothesis (*H*_0_)	Alternative hypothesis (*H*_1_)	Preference degree of rejection of *H*_0_	Test results
*Q*_*PM*_ ~ *Q*_*EN*_	*H*_0_: *Q*_*PM*_ ≤ *Q*_*EN*_	*H*_1_: *Q*_*PM*_ > *Q*_*EN*_	*P* = 1.56×10^-92^ < 0.05/2	*H=1*
*Q*_*PM*_ ~ *Q*_*CRITIC*_	*H*_0_: *Q*_*PM*_ ≤ *Q*_*CRITIC*_	*H*_1_: *Q*_*PM*_ > *Q*_*CRITIC*_	*P* = 1.02×10^-59^< 0.05/2	*H*=1
*Q*_*PM*_ ~ *Q*_*SD*_	*H*_0_: *Q*_*PM*_ ≤ *Q*_*SD*_	*H*_1_: *Q*_*PM*_ > *Q*_*SD*_	*P* = 8.16×10^-58^< 0.05/2	*H*=1
*Q*_*PM*_ ~ *Q*_*CCSD*_	*H*_0_: *Q*_*PM*_ ≤ *Q*_*CCSD*_	*H*_1_: *Q*_*PM*_ > *Q*_*CCSD*_	*P* = 1.48×10^-84^< 0.05/2	*H*=1

Based on the above simulation and test results, it can be concluded that the proposed method is the most suitable method to achieve high reliability solutions. In other words, compared to other methods, the proposed method can reveal the preferences of decision makers well and identify the solutions that decision makers prefer the most.

## 7. Conclusion

On the basis of ER method, a high reliability solution of MADA problem based on interval attribute weights and evaluation grade utility is proposed. The method deals with the following three cases:(1) the weight and utility of the interval attribute of the evaluation level;(2) interval attribute weight; (3) The interval utility of the evaluation level. This makes the method very flexible. Each case involves several iterations. In each iteration, the potential best alternatives are recognized and their ranking order is derived through computation of their spacing difference. Following the final iteration, an intact order is generated for the entire alternatives to serve as the solution for the MADA problem. The applicability and validity of CPA problem are verified by solving it with the proposed method.

In the subsequent research, we will extend the proposed method to the analysis of multi-attribute group decision problems, such as the extant group decision approaches.

## Supporting information

S1 Appendix Supplementary material of “A high reliability based evidential reasoning approach”. Appendix A. Distance between interval numbers. Appendix B. Original performance assessment of six cars. Appendix C. The methods of entropy, SD, CRITIC, and CCSD. Appendix D. The data of numerical analysis and the procedure of simulation experiment. (DOCX)
