# Supplementary material for: A high reliability based evidential reasoning approach
Source: PLoS One. 2025 May 19;20(5):e0317438. doi: 10.1371/journal.pone.0317438 (PMC12088526; doi:10.1371/journal.pone.0317438)
Supplement: S1 Appendix — (DOCX) [file pone.0317438.s001.docx]

**Supplementary material of “A high reliability based evidential reasoning approach”**

Yin Liu, Hao Li

Business School, Nanjing XiaoZhuang University, Nanjing 211171, China

^*^Corresponding author. Tel: 15656515237.

E-mail address: liuyin@njxzc.edu.cn(Y. Liu).

**Appendix A**

**Distance between interval numbers**

Given the application of distance between interval numbers into the criterion reliability determination upon involvement of incomplete assessments, the relevant concepts about such distance are described.

**Definition A.1.** [1] Let *E* be a non-empty collection of real numbers. Given *x* and *y* which enable *x*, *y* $\in$ *E*. Then the distance from *x* to *y*, which is denoted by *d*(*x*, *y*), satisfies

(reflexivity) *d*(*x*, *y*) = 0 if and only if *x* = *y*, (A.1)

(symmetry) *d*(*x*, *y*) = *d*(*y*, *x*), and (A.2)

(triangular inequality) *d*(*x*, *y*) ≤ *d*(*x*, *z*) + *d*(*z*, *y*), *z* $\in$ *E*. (A.3)

Apart from the foregoing properties, *d*(*x*, *y*) ≥ 0 is also a fundamental trait of distance between real numbers. On an assumption that the distances between arbitrary two points from two intervals (in addition to those from the interval intersection) are integrated into the inter-interval distance, Li et al. (2008) [2] gave the following definition of distance between interval numbers.

**Definition A.2.** [2] Given two interval numbers *a* = [*a*^-^, *a*^+^] and *b* = [*b*^-^, *b*^+^], suppose $\bar{a}$ = $0.5\cdot(a^{-}+a^{+})$, $\bar{b}$ = $0.5\cdot(b^{-}+b^{+})$, *l*(*a*) = $a^{+}-a^{-}$, *l*(*b*) = $b^{+}-b^{-}$, *c* = [*c*^-^, *c*^+^] = $a\cap b$, and *l*(*c*) = $c^{+}-c^{-}$, then the distance between *a* and *b* is defined as

*d*(*a*, *b*) = $\sqrt{I_{D}-I_{\cap}}$ (A.4)

= $\sqrt{\left(\bar{a}-\bar{b}\right)^{2}+\frac{1}{3}\cdot\left(\left(0.5\cdot l(a)\right)^{2}+\left(0.5\cdot l(b)\right)^{2}\right)-\frac{1}{6}\cdot\left(l(c)\right)^{2}}$, (A.5)

where *I_D_* = $\int_{0}^{1} \int_{0}^{1} \left((a^{-}+x\cdot l(a))-(b^{-}+y\cdot l(b))\right)^{2}dxdy$ and (A.6)

$I_{\cap}$ = $\int_{0}^{1} \int_{0}^{1} \left((c^{-}+x\cdot l(c))-(c^{-}+y\cdot l(c))\right)^{2}dxdy$. (A.7)

The distance *d*(*a*, *b*) satisfies the conditions shown in Definition A.1 [1]. Also, *d*(*a*, *b*) is larger than and equal to 0.

**Appendix B**

**Original performance assessment of six cars**

The performance assessment of six commercial vehicles is as shown in Table B.1. Most of the attributes in Table B.1 are related to the technical performances of a car. Suppose the performance of a car is classified into several categories (grades) like “Top”, “Excellent”, “Good”, “Average”, “Poor” and “Worst”. Define the following set of grades to assess car performance:

*Ω =* {*H_n_*, *n* = 1, …, 6} = {Worst, Poor, Average, Good, Excellent, Top}. (B.1)

All attributes may then be assessed with reference to this set of grades using the rule based information transformation technique. The detail transformation technique can be seen in Yang (2001) [3]. All attributes may then be assessed with reference to this set of grades using the rule based information transformation technique. To assess “acceleration”, for example, it is assumed that equivalence rules can be acquired. The first equivalence rule reads as follows:

1. If an executive car can accelerate from stand still to 60 mph in 7.4s, then as far as acceleration is concerned the car’s performance is at top level among the range of cars in question. This rule may be represented by a simple statement “If accelerating time is 7.4s, then performance is top”.

Other equivalence rules could be acquired in a similar way as follows:

2. If accelerating time is 7.8s, then performance is excellent.

3. If accelerating time is 8.2s, then performance is good.

4. If accelerating time is 8.7s, then performance is average.

5. If accelerating time is 9.2s, then performance is poor.

6. If accelerating time is 10s, then performance is worst.

The above equivalence rules are established for a range of executive cars in question. Other types of cars such as sport cars are not considered. Based on the rules, a set of evaluation grades for “acceleration” equivalent to *Ω* results as follows:

*Ω*^acceleration^ *=* {*H*_1_, *H*_2_, *H*_3_, *H*_4_, *H*_5_, *H*_6_} = {10, 9.2, 8.7, 8.2; 7.8; 7.4 } (B.2)

The transformation matrix for acceleration is given by

$\text{A}_{acceleration}= \begin{matrix} & 10 9.2 8.7 8.2 7.8 7.4 \\ \begin{matrix} worst \\ poor \\ average \\ good \\ excellent \\ top \end{matrix} & \left[\begin{matrix} 1 & 0 & 0 & 0 & 0 & 0 \\ 0 & 1 & 0 & 0 & 0 & 0 \\ 0 & 0 & 1 & 0 & 0 & 0 \\ 0 & 0 & 0 & 1 & 0 & 0 \\ 0 & 0 & 0 & 0 & 1 & 0 \\ 0 & 0 & 0 & 0 & 0 & 1 \end{matrix}\right] \end{matrix}$ (B.3)

Given the equivalence rules as shown in Eqs. (B.1)-(B.3). The data shown in Table B.1 can be transformed to belief distribution as shown in Table 3, so that all attributes are assessed in a unified format using the same set of evaluation grades as given by Eq. (B.1). Take the accelerating time (*h*_1_ = 8.8s) of car 1 for example. Since *H*_2_ = 9.2s and *H*_3_ = 8.7s and *H*_3_ < *h*_1_ < *H*_2_, we describe *h*_1_ = 8.8s as follows:

*B*(*e_i_*(*c*_1_)) = {(*H*_2,1_, *β*_2_(*c*_1_)), (*H*_3,1_, *β*_3_(*c*_1_))}, where *β*_2_(*c*_1_)) and *β*_3_(*c*_1_) are calculated as follows:

*β*_2_(*c*_1_)) = $\frac{H_{3,1}-h_{1}}{H_{3,1}-H_{2,1}}=\frac{8.7-8.8}{8.7-9.2}=0.2$, *β*_3_(*c*_1_) = 1− *β*_2_(*c*_1_) = 0.8. (B.4)

*B*(*e_i_*(*c*_1_)) can be equivalently represented using Eq. (B.4) where *β_n_*(*c*_1_) = 0 (*n* = 1, 4, 5, 6). Then, the assessments on Acceleration (*e*_1_) for *c*_1_ is calculated as

*B*(*e_i_*(*c*_1_)) = {(*H*_2,1_, 0.2), (*H*_3,1_, 0.8)} = {(Poor, 0.2), (Average, 0.8)} = {(P, 0.2), (A, 0.8))}, where P and A are abbreviated from “Poor” and “Average”, respectively. Other assessments can be transformed in the same way.

**Table B.1.** Original preference assessment of executive cars

| Performance | *c*_1_ | *c*_2_ | *c*_3_ | *c*_4_ | *c*_5_ | *c*_6_ |
| --- | --- | --- | --- | --- | --- | --- |
| Acceleration (*e*_1_) | 8.8 | 8.0 | 7.7 | 8.4 | 8.0 | 7.9 |
| Braking (*e*_2_) | 128 | 124 | 127 | 134 | 135 | 126 |
| Handling (*e*_3_) | B | A | B | B- | B+ | A |
| Horsepower (*e*_4_) | 196 | 152 | 182 | 183 | 138 | 171 |
| Ride quality (*e*_5_) | A- | B- | B | B+ | B+ | A- |
| Power train (*e*_6_) | B | B+ | A | B | A- | A |
| Fuel economy (*e*_7_) | 20 | 20 | 21 | 20 | 19 | 20 |

**Appendix C**

**The methods of entropy, SD, CRITIC, and CCSD**

(1) Attribute weight derivation by following the entropy process

For the objective production of attribute weights from a belief decision matrix as per the entropy process, entropy construction is necessary for individual alternative assessments on every attribute. For this purpose, we combine *B*(*e_i_*(*a_l_*)) (*i* = 1, …, *L*, *l* = 1, …, *M*) with *u*(*H_n_*) (*n* = 1, …, *N*) to produce the anticipated minimal and maximal utilities for alternative *a_l_* on attribute *e_i_* as follows

*u_min_*(*e_i_*(*a_l_*)) = $\sum_{n=2}^{N} \beta_{n,i}(a_{l})u(H_{n})$ + (*β*_1_,*_i_*(*a_l_*) + *β*_Ω_,*_i_*(*a_l_*))*u*(*H*_1_) and (C.1)

*u_max_*(*e_i_*(*a_l_*)) = $\sum_{n=1}^{N-1} \beta_{n,i}(a_{l})u(H_{n})$ + (*β_N_*,*_i_*(*a_l_*) + *β*_Ω_,*_i_*(*a_l_*))*u*(*H_N_*). (C.2)

Based on *u_min_*(*e_i_*(*a_l_*)) and *u_max_*(*e_i_*(*a_l_*)), the minimal satisfaction is formulated for an alternative *a_l_* on attribute *e_i_* as

*V*(*e_i_*(*a_l_*)) = $u_{\min}(e_{i}(a_{l}))-\max_{m\neq l}\{u_{\max}(e_{i}(a_{m}))\}$, (C.3)

where the value scope of *V*(*e_i_*(*a_l_*)) is [-1, 1]. The larger its value, the superior the foregoing *a_l_* on *e_i_*.

Then, normalized entropy is determined for *V*(*e_i_*(*a_l_*)) (*l* = 1, …, *M*) on the attribute *e_i_* (*i* = 1, ..., *L*)

*NE_i_* = $-\frac{\sum_{l=1}^{M} \tilde{V}(e_{i}(a_{l}))\ln\tilde{V}(e_{i}(a_{l}))}{\ln M}$, (C.4)

where $\tilde{V}(e_{i}(a_{l}))$ = $\frac{\bar{V}(e_{i}(a_{l}))}{\sum_{m=1}^{M} \bar{V}(e_{i}(a_{m}))}$ and $\bar{V}(e_{i}(a_{l}))$ = (*V*(*e_i_*(*a_l_*))-(-1))/2. Here, $\tilde{V}(e_{i}(a_{l}))$ and $\bar{V}(e_{i}(a_{l}))$ normalization is accomplished since *V*(*e_i_*(*a_l_*))$\in$[-1, 1]. *NE_i_* is restricted within [0,1] by denominator ln*M*. Next step is contrast intensity computation for the attribute *e_i_* (*i* = 1, ..., *L*) as

*CI_i_* = 1 - *NE_i_*. (C.5)

Hence, the weight of *e_i_* (*i* = 1, ..., *L*) is obtained as

*w_i_* = $\frac{CI_{i}}{\sum_{k=1}^{L} CI_{k}}$, *i* = 1, ..., *L*. (C.6)

(2) Attribute weight derivation by following the SD process

To obtain attribute weights objectively as per the SD procedure, computation of SD is accomplished for *V*(*e_i_*(*a_l_*)) (*l* = 1, …, *M*) on attribute *e_i_* (*i* = 1, ..., *L*) as

*σ_i_* = $\sqrt{\frac{1}{M}\sum_{l=1}^{M} (\bar{V}(e_{i}(a_{l}))-\frac{\sum_{m=1}^{M} \bar{V}(e_{i}(a_{m}))}{M})^{2}}$, (C.7)

where $\bar{V}(e_{i}(a_{l}))$ = (*V*(*e_i_*(*a_l_*))-(-1))/2 represents a normalization of *V*(*e_i_*(*a_l_*)) since *V*(*e_i_*(*a_l_*))$\in$[-1, 1]. Accordingly, weight is estimated for *e_i_* (*i* = 1, ..., *L*) as

*w_i_* = $\frac{\sigma_{i}}{\sum_{k=1}^{L} \sigma_{k}}$, *i* = 1, ..., *L*. (C.8)

(3) Attribute weight derivation by following the CRITIC process

For objective attribute weight obtainment as per the CRITIC procedure, an inter-attribute correlation coefficient matrix is formulated as

*r_ij_* = $\frac{C(B(e_{i}(\cdot)),B(e_{j}(\cdot)))}{\sqrt{E(B(e_{i}(\cdot)))\cdot E(B(e_{j}(\cdot)))}}$, *i*, *j* = 1, ..., *L*, (C.9)

where $C(B(e_{i}(\cdot)),B(e_{j}(\cdot)))$ = $\frac{1}{2}\sum_{l=1}^{M} (\sum_{n=1}^{N} \beta_{n,i}(a_{l})\cdot\beta_{n,j}(a_{l})+\beta_{\Omega,i}(a_{l})\cdot\beta_{\Omega,j}(a_{l}))$,

$E(B(e_{i}(\cdot)))$ = $\frac{1}{2}\sum_{l=1}^{M} (\sum_{n=1}^{N} \beta_{n,i}(a_{l})^{2}+\beta_{\Omega,i}(a_{l})^{2})$,

$E(B(e_{j}(\cdot)))$ = $\frac{1}{2}\sum_{l=1}^{M} (\sum_{n=1}^{N} \beta_{n,i}(a_{l})^{2}+\beta_{\Omega,j}(a_{l})^{2})$,

and $B(e_{i}(\cdot))$ represents the set of *B*(*e_i_*(*a_l_*)) (*l* = 1, ..., *M*).

Exploitation of Cauchy–Schwarz inequality yields $\sum_{l=1}^{M} \sum_{n=1}^{N} \beta_{n,i}(a_{l})\cdot\beta_{n,j}(a_{l})$≤ $\sum_{l=1}^{M} \sqrt{(\sum_{n=1}^{N} \beta_{n,i}(a_{l})^{2})\cdot(\sum_{n=1}^{N} \beta_{n,j}(a_{l})^{2})}$≤$\sqrt{(\sum_{l=1}^{M} \sum_{n=1}^{N} \beta_{n,i}(a_{l})^{2})\cdot(\sum_{l=1}^{M} \sum_{n=1}^{N} \beta_{n,j}(a_{l})^{2})}$, $\sum_{l=1}^{M} \beta_{\Omega,i}(a_{l})\cdot\beta_{\Omega,j}(a_{l})$≤$\sqrt{(\sum_{l=1}^{M} \beta_{\Omega,i}(a_{l})^{2})\cdot(\sum_{l=1}^{M} \beta_{\Omega,j}(a_{l})^{2})}$, and further $\sqrt{(\sum_{l=1}^{M} \sum_{n=1}^{N} \beta_{n,i}(a_{l})^{2})\cdot(\sum_{l=1}^{M} \sum_{n=1}^{N} \beta_{n,j}(a_{l})^{2})}$+$\sqrt{(\sum_{l=1}^{M} \beta_{\Omega,i}(a_{l})^{2})\cdot(\sum_{l=1}^{M} \beta_{\Omega,j}(a_{l})^{2})}$≤$\sqrt{(\sum_{l=1}^{M} (\sum_{n=1}^{N} \beta_{n,i}(a_{l})^{2}+\beta_{\Omega,i}(a_{l})^{2}))\cdot(\sum_{l=1}^{M} (\sum_{n=1}^{N} \beta_{n,j}(a_{l})^{2}+\beta_{\Omega,j}(a_{l})^{2}))}$, inferring that 0 ≤ *r_ij_* ≤ 1. Utilizing *r_ij_* of Eq. (B.9) and *σ_i_* of Eq. (B.7) (*i*, *j* = 1, ..., *L*), weight is determined for *e_i_* (*i* = 1, ..., *L*) as

*w_i_* = $\frac{\sigma_{i}\sum_{j=1}^{L} (1-r_{ij})}{\sum_{k=1}^{L} \sigma_{k}\sum_{j=1}^{L} (1-r_{kj})}$, *i* = 1, ..., *L*. (C.10)

(4) Attribute weight derivation by following the CCSD procedure

To objectively obtain attribute weights as per the CCSD procedure, the aggregated assessment is estimated for an alternative *a_l_* (*l* = 1, …, *M*) on the entire attributes aside from the *i*th one, i.e. *e_i_* (*i* $\in$ {1, ..., *L*}), as

$B^{i-}(a_{l})$ = ($\beta_{^{1}}^{i-}(a_{l})$, …, $\beta_{^{N}}^{i-}(a_{l})$) (*l* = 1, ..., *M*), (C.11)

With the utilization of ER algorithm, *B*(*e_j_*(*a_l_*)) (*j* = 1, …, *L*, *j* ≠ *i*) along with weight vector $w_{j}^{i-}$ = $\frac{w_{j}}{\sum_{k=1,k\neq i}^{L} w_{k}}$ (*j* = 1, ..., *L*, *j* ≠ *i*). Indubitably, constraint $\sum_{j=1,j\neq i}^{L} w_{j}^{i-}$=1 is fulfilled. The global ignorance degree of $B^{i-}(a_{l})$ is denoted by $\beta_{\Omega}^{i-}(a_{l})$ to fulfill $\sum_{n=1}^{N} \beta_{n}^{i-}(a_{l})$+$\beta_{\Omega}^{i-}(a_{l})$ = 1.

Similar to *r_ij_* in Eq. (B.9), the correlation coefficient of $B(e_{i}(\cdot))$ with $B^{i-}(\cdot)$, where the latter represents the set of $B^{i-}(a_{l})$ (*l* = 1, ..., *M*), is expressed as

*R_i_* = $\frac{C(B(e_{i}(\cdot)),B^{i-}(\cdot))}{\sqrt{E(B(e_{i}(\cdot)))\cdot E(B^{i-}(\cdot))}}$, *i* = 1, ..., *L*, (C.12)

where $C(B(e_{i}(\cdot)),B^{i-}(\cdot))$ = $\frac{1}{2}\sum_{l=1}^{M} (\sum_{n=1}^{N} \beta_{n,i}(a_{l})\cdot\beta_{_{n}}^{i-}(a_{l})+\beta_{\Omega,i}(a_{l})\cdot\beta_{_{\Omega}}^{i-}(a_{l}))$,

$E(B(e_{i}(\cdot)))$ = $\frac{1}{2}\sum_{l=1}^{M} (\sum_{n=1}^{N} \beta_{n,i}(a_{l})^{2}+\beta_{\Omega,i}(a_{l})^{2})$,

and $E(B^{i-}(\cdot))$ = $\frac{1}{2}\sum_{l=1}^{M} (\sum_{n=1}^{N} \beta_{_{n}}^{i-}(a_{l})^{2}+\beta_{_{\Omega}}^{i-}(a_{l})^{2})$.

It can be known that *R_i_* is also limited to [0, 1] in accordance with the process of verifying 0 ≤ *r_ij_* ≤ 1. Utilizing *σ_i_* of Eq. (B.7) (*i* = 1, ..., *L*) and *R_i_* of Eq. (B.12), the weight is estimated for attribute *e_i_* (*i* = 1, ..., *L*) through resolution of optimization model shown below:

MIN *J* = $\sum_{i=1}^{L} \left(w_{i}-\frac{\sigma_{i}\sqrt{1-R_{i}}}{\sum_{k=1}^{L} \sigma_{k}\sqrt{1-R_{k}}}\right)^{2}$ (C.13)

s.t. $\sum_{i=1}^{L} w_{i}$ = 1, (C.14)

*w_i_* ≥ 0, *i* = 1, ..., *L*. (C.15)

Note that ER analytical algorithm is implicitly included in this model. In addition, we can introduce the evaluation grade utility interval and related constraints into the optimization models extended by foregoing four processes.

**Appendix D**

**The data of numerical analysis and the procedure of simulation experiment**

**Table D.1.** Assessments of the twelve industries in the problem of selecting leading industries

| Attributes | *I*_1_ | *I*_2_ | *I*_3_ | *I*_4_ | *I*_5_ | *I*_6_ |
| --- | --- | --- | --- | --- | --- | --- |
| *e*_1_ | {(*G*, 0.1), (*E*, 0.9)} | {(*G*, 0.1), (*E*, 0.9)} | {(*A*, 0.1), (*G*, 0.5), (*E*, 0.4)} | {(*G*, 0.1), (*E*, 0. 9)} | {(*G*, 0.3), (*E*, 0.7)} | {(*G*, 0.2), (*E*, 0.8)} |
| *e*_2_ | {(*E*, 1)} | {(*A*, 0.2), (*G*, 0.5), (*E*, 0.3)} | {(*P*, 0.2), (*A*, 0.3), (*G*, 0.4), (*E*, 0.1)} | {(*A*, 0.2), (*G*, 0.4), (*E*, 0.4)} | {(*A*, 0.4), (*G*, 0.4), (*E*, 0.2)} | {(*A*, 0.6), (*G*, 0.1), (*E*, 0.3} |
| *e*_3_ | {(*E*, 1)} | {(*A*, 0.1), (*G*, 0.7), (*E*, 0.2)} | {(*A*, 0.1), (*G*, 0.2), (*E*, 0.7)} | {(*G*, 0.7), (*E*, 0.3)} | {(*A*, 0.1), (*G*, 0.8), (*E*, 0.1)} | {(*A*, 0.2), (*G*, 0.5), (*E*, 0.3)} |
| *e*_4_ | {(*W*, 0.2), (*P*, 0.8)} | {(*P*, 0.2), (*A*, 0.4), (*G*, 0.3), (*E*, 0.1)} | {(*P*, 0.1), (*A*, 0.3), (*G*, 0.4), (*E*, 0.2)} | {(*W*, 0.3), (*P*, 0.5), (*A*, 0.2)} | {(*A*, 0.1), (*G*, 0.8), (*E*, 0.1)} | {(*A*, 0.6), (*G*, 0.3), (*E*, 0.1)} |
| *e*_5_ | {(*G*, 0.4), (*E*, 0.6)} | {(*A*, 0.1), (*G*, 0.3), (*E*, 0.6)} | {(*A*, 0.2), (*G*, 0.4), (*E*, 0.4)} | {(*G*, 0.1), (*E*, 0.9)} | {(*G*, 0.6), (*E*, 0.4)} | {(*G*, 0.2), (*E*, 0.8)} |
| *e*_6_ | {(*W*, 0.8), (*P*, 0.2)} | {(*W*, 0.3), (*P*, 0.4), (*A*, 0.3)} | {(*P*, 0.3), (*A*, 0.5), (*G*, 0.1), (*E*, 0.1)} | {(*W*, 0.4), (*P*, 0.5), (*A*, 0.1)} | {(*A*, 0.2), (*G*, 0.2), (*E*, 0.6)} | {(*W*, 0.3), (*P*, 0.6), (*A*, 0.1)} |
| *e*_7_ | {(*W*, 0.6), (*P*, 0.3), (*A*, 0.1)} | {(*P*, 0.3), (*A*, 0.1), (*G*, 0.4), (*E*, 0.2)} | {(*W*, 0.5), (*P*, 0.4), (*A*, 0.1)} | {(*P*, 0.3), (*A*, 0.3), (*G*, 0.2), (*E*, 0.2)} | {(*A*, 0.1), (*G*, 0.6), (*E*, 0.3)} | {(*P*, 0.3), (*A*, 0.1), (*G*, 0.4), (*E*, 0.2)} |

**Table D.1.** Assessments of the twelve industries in the problem of selecting leading industries (continued)

| Attributes | *I*_7_ | *I*_8_ | *I*_9_ | *I*_10_ | *I*_11_ | *I*_12_ |
| --- | --- | --- | --- | --- | --- | --- |
| *e*_1_ | {(*G*, 0.5), (*E*, 0.5)} | {(*G*, 0.3), (*E*, 0.7)} | {(*G*, 0.3), (*E*, 0.7)} | {(*A*, 0.1), (*G*, 0.1), (*E*, 0.8)} | {(*G*, 0.1), (*E*, 0.9)} | {(*A*, 0.1), (*G*, 0.2), (*E*, 0.7)} |
| *e*_2_ | {(*G*, 0.5), (*E*, 0.5)} | {(*G*, 0.2), (*E*, 0.8)} | {(*G*, 0.3), (*E*, 0.7)} | {(*G*, 0.5), (*E*, 0.5)} | {(*G*, 0.3), (*E*, 0.7)} | {(*A*, 0.2), (*G*, 0.6), (*E*, 0.2)} |
| *e*_3_ | {(*A*, 0.1), (*G*, 0.5), (*E*, 0.4)} | {(*G*, 0.9), (*E*, 0.1)} | {(*W*, 0.4), (*P*, 0.6)} | {(*A*, 0.1), (*G*, 0.5), (*E*, 0.4)} | {(*G*, 0.6), (*E*, 0.4)} | {(*A*, 0.2), (*G*, 0.5), (*E*, 0.3)} |
| *e*_4_ | {(*W*, 0.2), (*P*, 0.5), (*A*, 0.3)} | {(*W*, 0.3), (*P*, 0.5), (*A*, 0.2)} | {(*G*, 0.3), (*E*, 0.7)} | {(*W*, 0.3), (*P*, 0.5), (*A*, 0.2)} | {(*P*, 0.1), (*A*, 0.5), (*G*, 0.3), (*E*, 0.1)} | {(*W*, 0.2), (*P*, 0.6), (*A*, 0.2)} |
| *e*_5_ | {(*A*, 0.8), (*G*, 0.1), (*E*, 0.1)} | {(*A*, 0.1), (*G*, 0.3), (*E*, 0.6)} | {(*G*, 0.4), (*E*, 0.6)} | {(*G*, 0.3), (*E*, 0.7)} | {(*A*, 0.1), (*G*, 0.7), (*E*, 0.2)} | {(*G*, 0.5), (*E*, 0.5)} |
| *e*_6_ | {(*W*, 0.5), (*P*, 0.4), (*A*, 0.1)} | {(*W*, 0.1), (*P*, 0.7), (*A*, 0.2)} | {(*G*, 0.6), (*E*, 0.4)} | {(*P*, 0.4), (*A*, 0.3), (*G*, 0.2), (*E*, 0.1)} | {(*W*, 0.6), (*P*, 0.3), (*A*, 0.1)} | {(*W*, 0.5), (*P*, 0.2), (*A*, 0.3)} |
| *e*_7_ | {(*P*, 0.5), (*A*, 0.3), (*G*, 0.1), (*E*, 0.1)} | {(*W*, 0.4), (*P*, 0.2), (*A*, 0.4)} | {(*A*, 0.2), (*G*, 0.5), (*E*, 0.3} | {(*W*, 0.5), (*P*, 0.3), (*A*, 0.2)} | {(*W*, 0.6), (*P*, 0.4)} | {(*W*, 0.3), (*P*, 0.4), (*A*, 0.3)} |

**Table D.2.** Process of generating the reliability of the aggregated solution from random decision matrices by using the four representative methods.

| **Simulation experiment:** Determining 600 sets of attribute weights with the four representative methods and the corresponding 600 sets of reliability of the aggregated solution. |
| --- |
| **Input:** (1) The 600 random belief decision matrices $B(e_{i}(a_{l}))_{L\times L}^{\text{T}}$ (*L* = 5, …, 10, *T* = 1, …, 100, *i*, *l* =1, …, *L*,) for the six combinations of alternatives and attributes in Table 9 are generated. (2) The utilities of assessment grades are provided, that is, *u*(*H_n_*)) (*n* = 1, …, 5) = (0, 0.25, 0.5, 0.75, 1).  **Output:** The 600 sets of attribute weights $w_{i}^{(T, t)}$ (*T* = 1, …, 100, *t* = 1, …, 5, *i* = 1, …, *L*,) and the corresponding 600 sets of reliability of the aggregated solution generated by using the four representative methods are obtained.  **Step 1:** The stochastic simulation is used to generate 600 decision matrices for the six combinations of alternatives and attributes.  **Step 2:** The four typical methods and the proposed method are applied to generate the 600 sets of attribute weights.  **Step 3:** The 600 sets of assessments are combined to generate the 600 sets of aggregated assessment by using the ER analytical algorithm and the 600 sets of attribute weights in Step 2, which are further combined to produce the 600 sets of expected utilities with *u*(*H_n_*).  **Step 4:** Based on the 600 sets of expected utilities *u*(*a_l_*)(*^T^*, *^t^*), the 600 sets of minimal satisfaction of the alternative are obtained.  **Step 5:** By using the obtained 600 sets of minimal satisfaction of alternative in Step 5, the 600 sets of reliability of the aggregated solution of the four representative methods and the proposed method are obtained. |

**Reference**

1. Irpino A, Verde R, Dynamic clustering of interval data using a wasserstein-based distance. Pattern Recognition Letters. 2008; 29 (11): 1648-1658.
2. Li X, Zhang SL, et al. Rank of Interval Numbers Based on a New Distance Measure. Journal of Xihua University Natural Science. 2008.
3. Yang JB. Rule and utility based evidential reasoning approach for multiattribute decision analysis under uncertainties.2001; 1(131): 31-61.
